# A universal polymer shell-isolated nanoparticle (SHIN) design for single particle spectro-electrochemical SERS sensing using different core shapes†

**DOI:** 10.1039/d1na00473e

**Published:** 2021-09-21

**Authors:** Delali K. Boccorh, Peter A. Macdonald, Colm W. Boyle, Andrew J. Wain, Leonard E. A. Berlouis, Alastair W. Wark

**Affiliations:** Centre for Molecular Nanometrology, Technology and Innovation Centre, Dept. of Pure & Applied Chemistry, University of Strathclyde 99 George St Glasgow G1 1RD UK alastair.wark@strath.ac.uk +44 (0)141 548 3084; National Physical Laboratory Hampton Road Teddington TW11 0LW UK; Dept. of Pure & Applied Chemistry, University of Strathclyde 295 Cathedral St Glasgow G1 1XL UK

## Abstract

Shell-isolated nanoparticles (SHINs) have attracted increasing interest for non-interfering plasmonic enhanced sensing in fields such as materials science, biosensing, and in various electrochemical systems. The metallic core of these nanoparticles is isolated from the surrounding environment preventing direct contact or chemical interaction with the metal surface, while still being close enough to enable localized surface plasmon enhancement of the Raman scattering signal from the analyte. This concept forms the basis of the shell isolated nanoparticle-enhanced Raman spectroscopy (SHINERS) technique. To date, the vast majority of SHIN designs have focused on SiO_2_ shells around spherical nanoparticle cores and there has been very limited published research considering alternatives. In this article, we introduce a new polymer-based approach which provides excellent control over the layer thickness and can be applied to plasmonic metal nanoparticles of various shapes and sizes without compromising the overall nanoparticle morphology. The SHIN layers are shown to exhibit excellent passivation properties and robustness in the case of gold nanosphere (AuNP) and anisotropic gold nanostar (AuNS) core shapes. In addition, *in situ* SHINERS spectro-electrochemistry measurements performed on both SHIN and bare Au nanoparticles demonstrate the utility of the SHIN coatings. Correlated confocal Raman and SEM mapping was achieved to clearly establish single nanoparticle SERS sensitivity. Finally, confocal *in situ* SERS mapping enabled visualisation of the redox related molecular structure changes occurring on an electrode surface in the vicinity of individual SHIN-coated nanoparticles.

## Abstract 2

A new design of shell isolated nanoparticles (SHINs) is described that can be applied to various gold core shapes for non-interfering surface-enhanced Raman scattering (SERS) sensing at single nanoparticle resolution in electroactive environments.

## Graphical abstract

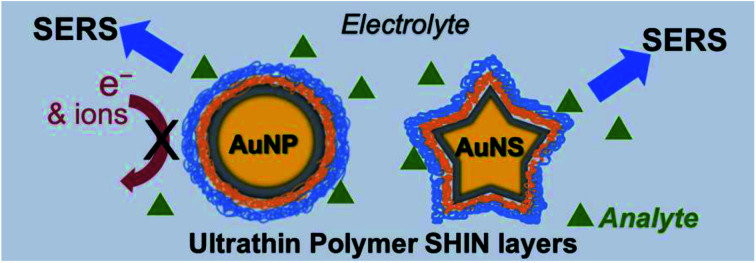

## 1. Introduction

Shell-isolated nanoparticles (SHINs) are a class of nanomaterials featuring an inert dielectric shell that physically isolates an inner core metallic particle from the surrounding environment.^1^ Of particular interest is the application of SHINs for surface-enhanced Raman scattering (SERS), where the plasmonic nanoparticle core is coated with a sufficiently thin protective layer that only slightly impairs the enhancement of analyte Raman scattering signals while providing an effective barrier that prevents the plasmonic metallic core from interacting directly (*e.g.* redox and catalytic processes) with the measurement environment. Since this method, known as shell-isolated nanoparticle-enhanced Raman spectroscopy (SHINERS), was introduced in 2010,^2^ it has been successfully utilized for studying the electrochemical response of molecules on the surfaces of single crystal electrodes^3–5^ for *in situ* tracking of catalytic processes,^6^ and has found other applications in biosensing, materials science, catalysis, and environmental safety.^7–11^

The vast majority of SHINERS studies reported in the literature to date have focused on a thin silica (SiO_2_) shell surrounding a spherical gold or silver plasmonic nanoparticle core. In contrast, thicker SiO_2_ shells around metallic nanoparticles have been more widely researched for applications such as for SERS nanotags.^12,13^ However, challenges with silica coating of colloidal systems including hydrolysis susceptibility and the subsequent presence of pinholes in thin layers,^14^ inflexible control over coating thickness,^15^ and degradation of the silica layer over time^16,17^ have encouraged the study of alternative SHIN designs.^18^ Dielectric shell materials such as Al_2_O_3_,^19^ MnO_2_,^20^ TiO_2_,^21^ and ZrO_2_ ^22^ have been applied to plasmonic particles for specific sensing functions in recent years. Despite the widespread use of organic polymers (*e.g.* self-assembling alkanethiols,^23^ polyelectrolytes,^24–26^ biomolecules^27^) on colloidal metallic surfaces, there is only one example (to our knowledge) of a passivating polymer-based SHINERS system where an ultrathin layer of polydopamine (PDA) was incorporated onto gold nanoparticles by Ye *et al.*^28^ and applied for the detection of benzotriazole. However, PDA is redox-active due to an abundance of catechol groups^29,30^ and allows both anionic and cationic species to migrate depending on solution pH,^31^ which restricts the potential windows within which such coatings could be used without interference. For example, it has been shown that PDA deposited in the form of a nanofilm exhibits limited resistance to electron transfer processes.^32^

In addition, while the plasmonic enhancement of SERS measurements associated with anisotropic nanoparticle shapes compared to nanospheres has been widely reported,^33,34^ the application of SHINs using non-spherical core shapes is limited. Examples of SiO_2_ coatings for anisotropic SHINs include nanorods,^35–37^ nanocubes,^36,38,39^ and only one recent example involving nanostars,^40^ where the isolating properties of the surrounding silica layer are demonstrated. More generally, there are a few examples of silica coated nanostars used in SERS applications, although controlling the formation of the silica layer to conform to the particle shape, which is crucial for SERS analytes to access hotspots at the tips of nanostar branches, remains difficult.^41–43^

In this article, we undertake the challenge of developing a new SHIN layer design for SERS applications which circumvents the difficulties associated with SiO_2_ coating and can be universally applied to a variety of gold nanoparticle sizes and shapes. To achieve this, a robust method for the multi-layer self-assembly of an alkanethiol and two separate polymers was developed and demonstrated on both quasi-spherical gold nanoparticles (AuNPs) and gold nanostars (AuNSs). A variety of spectroscopic and electrochemical measurements were performed in both cases to characterize the isolating properties of the SHIN layers. Furthermore, using methylene blue (MB); which is a popular biological stain and redox indicator,^44^ as well as being an important benchmark compound to determine catalytic activity;^45^ the SHINERS performance of both the AuNP and AuNS SHINs was demonstrated using *in situ* confocal SERS mapping of an electrode surface under an applied potential. These experiments highlight the utility of our approach for the non-interfering analysis of electrochemical processes at spatial resolutions approaching the single nanoparticle level.

## 2. Experimental

### 2.1 Materials

The following chemicals were all sourced from Sigma Aldrich and used without modification or purification unless stated otherwise: trisodium citrate dihydrate (Na_3_C_6_H_5_O_7_), chloroauric acid (HAuCl_4_·3H_2_O), l-ascorbic acid (C_6_H_8_O_6_), silver nitrate (AgNO_3_), sodium chloride (NaCl), sodium hydroxide (NaOH), 11-mercaproundecanoic acid (C_11_H_22_O_2_S), methylene blue (C_16_H_18_ClN_3_S), pyridine anhydrous (C_5_H_5_N), 4,4′-bipyridine (C_10_H_8_N_2_), polystyrene sulfonate (PSS) ∼10 000 MW, and indium tin oxide (ITO) coated glass slides (surface resistivity of 30–60 Ω, 1-inch square). Poly(diallyl dimethylammonium chloride) (PDDA) ∼8500 MW was sourced from Polysciences Europe. All glassware and PTFE-coated stirrer bars used for nanoparticle synthesis experiments were soaked for at least 2 hours in aqua regia before being rinsed with water several times. Millipore MilliQ purified water was used in all experiments.

### 2.2 Gold nanosphere (AuNP) (∼80 nm) preparation

Synthesis was adapted from Leng *et al.*^46^ Seed synthesis: citrate capped AuNPs seeds were synthesised by first adding 93 mL of water to a clean 250 mL conical flask containing 9.85 mg HAuCl_4_·3H_2_O (0.25 mM). A clean stirrer bar was then added to the flask, which was then heated on a hotplate until boiling under vigorous stirring. Next, 25.7 mg of Na_3_C_6_H_5_O_7_ (0.875 mM) dissolved in 7 mL of water was added dropwise to the flask. This mixture of reagents corresponds to a Au : citrate molar ratio of 1 : 3.5. The contents of the flask were then left to boil under vigorous stirring for 15 minutes before being allowed to cool. The solution was then centrifuged (4000 relative centrifugal force (RCF)) in 50 mL tubes for 45 minutes and resuspended in water to remove excess citrate. See Fig. S1† for a UV-Vis spectrum of the nanoparticle seeds. Growth solution: larger AuNPs were obtained by adding 2 mL of the citrate capped seeds (extinction ∼1.38) to a 250 mL conical flask containing 80 mL of water. HAuCl_4_·3H_2_O (0.25 mM) and Na_3_C_6_H_5_O_7_ (0.375 mM) were then added, and the mixture stirred at a moderate speed for 3 hours. The colloid was then split equally between two 50 mL centrifuge tubes (40 mL in each) and centrifuged (500 RCF) for 1 hour. The clear supernatant was then decanted, and the remaining nanoparticle pellet in each tube was resuspended in 20 mL of water to increase particle concentration.

### 2.3 Gold nanostar (AuNS) preparation

A surfactant-free synthesis method was adapted from Indrasekara *et al.*^34^ Seed synthesis: the same seed solution as described above for the AuNP preparation was used. Growth solution: 40 μL of HAuCl_4_·3H_2_O (125 mM) was diluted in 20 mL Milli-Q water. The solution was then stirred at medium speed and 4 μL of 5 M HCl was added. This was followed by the addition of 200 μL of 3 mM AgNO_3_. The reaction mixture was then left to stir for a minute before 100 μL of the citrate capped seed solution was added followed directly by 400 μL of 100 mM ascorbic acid. A colour change occurred immediately in which the pale-yellow solution instantly turned a dark blue colour. The colloid was then placed into 1.5 mL centrifuge tubes and centrifuged at 270 RCF for 30 minutes. The centrifuged AuNS were then used on the same day for SHIN preparation. In contrast to the bare nanostars, the SHIN coated nanostars were stable for at least one month.

### 2.4 SHIN fabrication

The same procedures were applied for both AuNP and AuNS particles unless stated otherwise. 11-Mercaptoundecanoic acid (MUA) functionalisation: 1 mL of seed grown AuNPs (optical density ∼1.5 and 1.2 for AuNP and AuNS, respectively) were distributed in 1.5 mL centrifuge tubes and the pH of the AuNP suspension in each tube was adjusted to around 10 using 0.5 M NaOH (∼20 μL). MUA in ethanol (0.1 mL, 10 mM) was then added to each centrifuge tube which were then placed in a shaker at around 750 rpm for 1 hour. The centrifuge tubes were then removed from the shaker and centrifuged (270 RCF) for 30 minutes and resuspended in water adjusted to pH 10 using NaOH. AuNS solutions were not centrifuged at this stage to avoid aggregation. Polyelectrolyte coating: 0.25 mL of PDDA (35 μL mL^−1^ water) was then added to each centrifuge tube containing 1 mL of AuNP@MUA and placed in a shaker for 10 minutes. The tubes were centrifuged (214 RCF) for 15 minutes and resuspended in water (1 mL). Next, 0.25 mL of PSS solution (10 mg mL^−1^ water) was added to each centrifuge tube containing 1 mL of AuNP@MUA@PDDA placed in a shaker for 10 minutes. The tubes were centrifuged at 172 RCF for 15 minutes and resuspended in water (1 mL). This resulted in a final AuNP@MUA@PDDA@PSS (referred to hereafter as “AuNP SHIN”) solution.

### 2.5 Nanoparticle characterisation

UV-Vis analysis was performed using an Agilent Cary 60 spectrophotometer. A Malvern Zetasizer instrument was used for zeta potential measurements with each measurement performed in triplicate and averaged. For scanning electron microscopy (SEM) measurements, a FEI Quanta 250 FEG-ESEM field environmental SEM instrument operating in high vacuum mode was used to obtain nanoparticle images. Rayleigh-based image and tracking analysis was performed using a Nanosight LM10 instrument. Nanoparticle Tracking Analysis (NTA) was performed on Nanosight software version 2.0. Videos up to 2 min long captured using the LM10 (Rayleigh scattering) were analysed using the NTA software.

### 2.6 Cyclic voltammetry characterisation of SHIN layers

All cyclic voltammetry (CV) measurements were performed on a Solartron Analytical Potentiostat. All bulk measurements were performed using a platinum counter electrode, and a fritted Ag/AgCl reference electrode (in saturated KCl) at a scan rate of 50 mV s^−1^, while nanoscale measurements were performed using a saturated calomel electrode (SCE) as the reference. Working electrodes used are described below.

To determine whether the polyelectrolyte layers used in the SHIN design undergo redox reactions in the aqueous potential window, solutions of PDDA (35 μL mL^−1^) and PSS (10 mg mL^−1^) were prepared by dissolving each in 5 mM NaCl. For each polyelectrolyte solution, ITO glass was immersed in the test solution for 30 minutes before being removed and allowed to dry. CV measurements were then carried out separately on each coated ITO electrode in 0.5 M KCl.

To test the passivation effectiveness of the SHIN layers on the bulk scale; MUA, PDDA, and PSS were immobilised on a bulk gold rotating disk electrode (electrode area 0.38 cm^2^) by immersing the electrode sequentially into solutions of MUA in ethanol (10 mM), PDDA in 5 mM NaCl (35 μL mL^−1^), and PSS in 5 mM NaCl (10 mg mL^−1^). Before SHIN layer immobilisation, the electrode was polished with silica powder. The electrode was immersed for 12 hours in MUA solution, followed by 1 hour each in PDDA and PSS solutions. After each step, the electrode was gently washed with water and dried under nitrogen. CV was then carried out in a solution containing 1 mM K_4_[Fe(CN)_6_] in 0.1 M KCl.

To examine the passivation effectiveness of the completed nanoparticle SHINs, each SHIN sample to be analysed was immobilised on an ITO-coated glass slide by pipetting a small volume onto the electrode surface. The SHIN sample was then left to dry on the ITO electrode surface before analysis. CV measurements were then carried out in 0.1 M H_2_SO_4_ at a scan rate of 50 mV s^−1^. The current density was calculated using the geometric area of the ITO working electrode (6.45 cm^2^).

### 2.7 Correlated confocal Raman and SEM mapping

An ITO-coated glass slide was utilised to obtain correlated Raman and SEM maps. The slide was prepared by washing with acetone, followed by methanol in an ultrasonic bath then dried under nitrogen before being placed in an oxygen plasma cleaner (Diener Femto low pressure plasma system) for 3 minutes to create a hydrophilic, negatively charged surface. PDDA (10 mg mL^−1^), dissolved in aqueous 5 mM NaCl was then pipetted onto the surface and left for 10 minutes before being rinsed off thoroughly with water and dried with a nitrogen stream resulting in a stable positively charged surface. SHIN particles (OD ∼1) were then immobilised on the ITO slide by pipetting a small amount into the surface and allowing the solution to sit for 1 hour in a humidity chamber to achieve a low particle density on the surface and prevent drying induced aggregation. The slide was then rinsed with water and again dried under nitrogen. A solution of 0.1 μM methylene blue was then pipetted onto the surface and left to sit for at least 30 minutes before being rinsed with water and dried under nitrogen. Finally, a metallic TEM reference grid (400 mesh, H7 Maxtaform) was immobilized on to the ITO surface with nail varnish.

Raman mapping was carried out utilising a WITEC Alpha300 instrument employing a 633 nm excitation laser. The maps were obtained using a 100× Olympus MPlan objective (NA 0.9) and data processing was performed using WITEC project 2.1 software. The reference grid was used to aid the location correlation on both the Raman and SEM instruments. The SEM images were obtained on a FEI Quanta 250 FEG-ESEM field environmental SEM instrument operating in high vacuum mode.

### 2.8 Spectro-electrochemical measurements

ITO-coated glass slides were used as the working electrodes and prepared in the same manner as described in Section 2.7 above. For some experiments, the ITO working electrode was spatially partitioned into two areas *via* silicone rubber wells to enable side-by-side SERS comparison of the different particles. The nanoparticle colloids to be analysed were then pipetted onto the ITO surface and left for up to 1 hour in a humid environment to achieve a relatively low-density surface coating and prevent the particle solution from drying (and so, inducing aggregation) in the wells. The ITO slide was then rinsed and dried under a stream of nitrogen. *In situ* SERS was performed using a WITEC Alpha300 system utilising a 633 nm laser and a Nikon NIR Apo 60× (NA 1.0) water dipping objective. The corresponding laser powers and integration times are stated alongside the data. The custom Perspex cell design is shown in Fig. S2† with a Ag/AgCl wire reference electrode (stored in saturated KCl) and an Au wire counter electrode used alongside the ITO working electrode. Potentiostatic control was achieved by a PalmSens4 instrument, manufactured by PalmSens BV.

Colour dark-field images of the ITO electrode surface were acquired using a Nikon Eclipse LV100 microscope in transmission equipped with an oil dark-field condenser, a 50× (NA 0.55) ELWD BD objective and a Nikon D50 camera.

## 3. Results and discussion

### 3.1 Design of new SHIN substrate

**Scheme 1:**
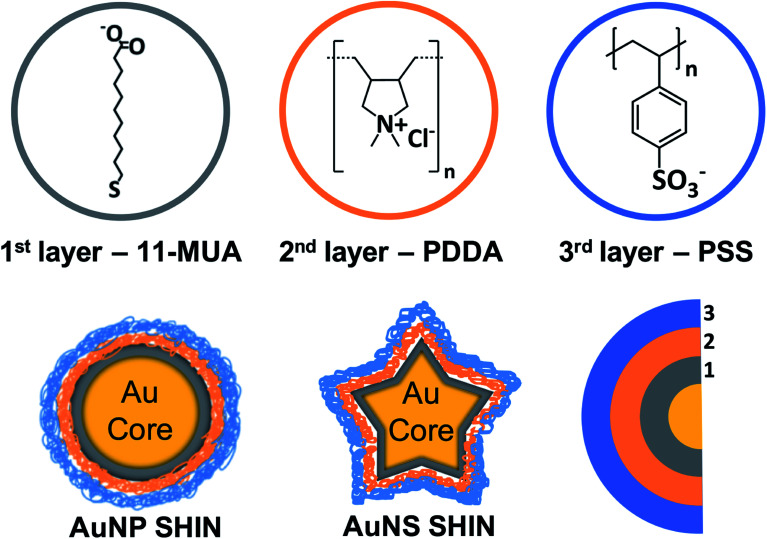
Schematic overview of SHIN layer design that was applied to both gold nanosphere and nanostar core shapes with the molecular structures of MUA, PDDA, and PSS represented.

An overview of the SHIN design that can be readily applied to a variety of nanoparticles shapes and sizes is shown in Scheme 1. Our approach was based on achieving a number of key requirements for SHIN layers: (i) surface passivation of the gold surface, (ii) stability and aqueous solubility, (iii) excellent control of the overall layer(s) thickness, (iv) a robust method for preparation, (v) the scope to tune the particle surface chemistry, and (vi) enabling application for both anisotropic as well as spherical shapes. MUA was selected as the base layer due to the molecule's tendency to form a densely-packed self-assembled monolayer (SAM) layer on gold,^47^ which has the ability to completely passivate an electrode surface thus preventing any electrochemical reactions.^48^ The polyelectrolytes, PDDA and PSS, were chosen as they allow a layer by layer (LbL) approach to be utilised due to their opposite polarities, which leads to the formation of ultrathin, hydrophilic layers. This approach allows the synthesised SHINs to prevent migration of both cations and anions to the gold core while remaining suitable for use in complex, biologically relevant environments.^49–51^ To assess whether the SHIN layer candidates would provide sufficient passivation, CV was performed after each layer was sequentially immobilised on a bulk gold electrode surface. The MUA, PDDA and PSS layers were then applied to two different sizes of spherical nanoparticles (∼40 nm and ∼80 nm diameter) as well as gold nanostars (∼120 nm).

The development of the SHIN layers primarily focused on the larger nanocolloid substrates (*i.e.* ≥80 nm) as initial studies on smaller AuNPs (∼40 nm) demonstrated a lower plasmonic enhancement of the SERS signal. This approach is supported by several previous SERS studies at single particle level using similarly large AuNPs.^52,53^ Although the SERS enhancement of gold nanostars is well-established,^54^ there are very few studies on SHIN coated nanostars.^40^

Bulk characterization of individual SHIN layers: prior to the fabrication of the SHIN particles, it was necessary to assess the electrochemical properties of the chosen insulating layers. CV measurements were performed to probe whether the polymer layers were redox-active within the aqueous solvent potential window. The voltammograms of PDDA and PSS adsorbed on ITO electrodes shown in Fig. S3† demonstrate that neither polymer undergoes any redox reactions between −1 V and 1 V *vs.* Ag/AgCl as indicated by the absence of any reduction or oxidation peaks.

#### Surface passivation

Further CV experiments were performed in the presence of 1 mM ferrocyanide in 0.5 M KCl electrolyte with each SHIN layer self-assembled onto the Au disc working electrode surface. The voltammograms in Fig. S4† show that the reversible Fe(CN)_6_^4−^/Fe(CN)_6_^3−^ redox peaks are completely suppressed when a packed MUA monolayer was allowed to form overnight on the Au electrode surface. Subsequent additions of the polyelectrolyte layers of PDDA and PSS, corresponding to the complete SHIN design did not interfere with the passivation provided by the MUA.

### 3.2 SHIN fabrication

**Fig. 1:**
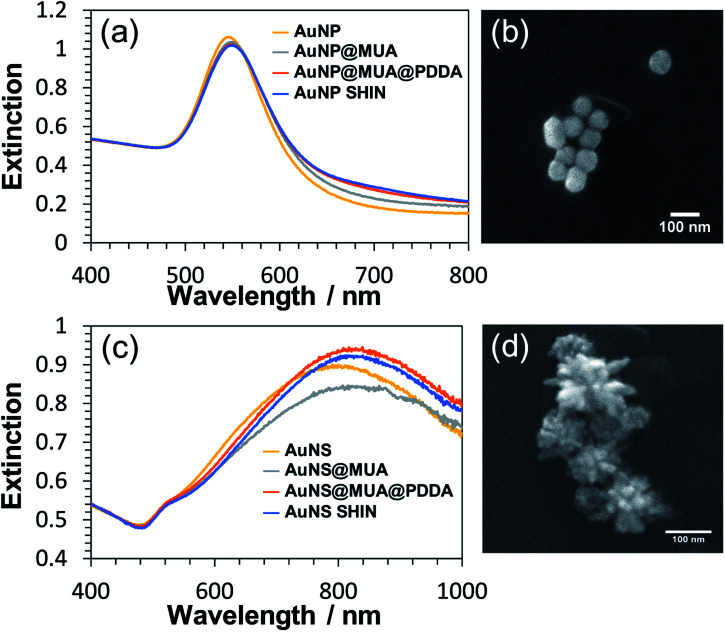
UV-Vis characterization of (a) AuNP and (c) AuNS SHIN fabrication. The spectra in each data series were normalised at 450 nm for comparison. Representative SEM images of the AuNP SHIN and the AuNS SHIN particles are shown in (b) and (d) with a scale bar of 100 nm in each.

Each step of the LbL SHIN fabrication process on both the nanosphere and nanostar particle substrates was monitored using UV-Vis spectroscopy, with the results shown in Fig. 1 alongside representative SEM images of both nanoparticle shapes. Additional SEM data is also shown in the ESI† (Fig. S5 and S6). For the bare spherical nanoparticles (Fig. 1(a)), the localised surface plasmon resonance (LSPR) *λ*_max_ was observed at 545 nm, which subsequently red-shifted to 549 nm upon the formation of the MUA monolayer. It was important during this step to adjust the pH of the colloidal solution to ∼ pH 10 to ensure that the carboxyl end groups of MUA were extensively deprotonated so as to maintain the colloidal stability *via* electrostatic repulsion. This was supported by the measured zeta-potentials of −44 mV and −36 mV for the initial citrate-coated AuNP stock and the AuNP@MUA particles, respectively. Subsequent changes in the zeta-potentials to +50 mV and −69 mV for the AuNP@MUA@PDDA and AuNP@MUA@PDDA@PSS (*i.e.* AuNP SHIN) particles respectively indicate successful coatings, with the opposing charges reflecting the polarity of the outermost polymer layer. The zeta-potential data is summarized in Table S1† and nanoparticle tracking analysis (NTA) data is shown in Fig. S7.† No evidence of nanoparticle aggregation was observed in the video tracking and it is interesting to note an increase of ∼5 nm in the average hydrodynamic diameter of the diffusing particles as well as a broadening of the size distribution when MUA is the outer layer. This can be attributed to the alkane backbone of MUA increasing the hydrophobicity of the particle when suspended in water with the resulting changes in the surrounding solvent layer contributing to a reduced rate of Brownian motion and so, the larger NTA size approximation. This is then reversed when the subsequent polyelectrolyte layers are added.

For the bare Au nanostar particles (Fig. 1(c)), the LSPR *λ*_max_ was observed at 790 nm and features a broad LSPR peak profile as expected.^34,55^ The trend in the measured zeta potential for the AuNS SHINs (summarised in Table S2†) was consistent with that observed for the AuNPs. Evidence for the successful LbL coatings is again provided by the zeta potential data, highlighting large negative, positive, and negative particle surface potentials upon addition of the MUA, PDDA, and PSS layers, respectively. The self-assembly of alkanethiol monolayers as well as the LbL assembly of polyelectrolytes around gold nanoparticles is well-established with TEM^56^ and analytical ultracentrifugation^26^ studies indicating layer thicknesses on the order of 1–2 nm. Furthermore, in this work no salt was added during the polyelectrolyte assembly which also promotes thinner layers compared to typical procedures reported in the literature utilizing several mM (or higher) of added salt. The combination of high zeta-potential values and improved colloidal stability confirm that an effective polyelectrolyte coating has been introduced.

The synthesis of the SHIN particles was found to be very reproducible and key to their successful preparation is the control of the solution pH during the MUA adsorption step. After subsequent polyelectrolyte coatings, the solution pH can then be adjusted over a wide range. With the passivating layers, the colloid was afforded increased colloidal stability, as corroborated in the literature.^57–59^ Fig. S8† compares UV-Vis spectra of repeated syntheses for the same core AuNP sample, showing very minor differences in the resulting spectra depending on the storage time of the core particle solution. It was also found that the SHIN fabrication could be easily adapted to various citrate-based colloids of different sizes. Fig. S9† highlights the long-term stability of the AuNP SHIN particles with very little change in the UV-Vis spectrum observed after over a year of storage with any sedimentation easily reversed on agitation with no significant aggregation. The impact of this increased stability was especially apparent when comparing AuNS SHINs to bare AuNS stock, as the highly anisotropic nanostars typically continue to change in morphology in the days after synthesis while the SHIN coated samples will remain more stable (see Fig S10†).

### 3.3 Electrochemical characterisation of SHIN particles

In order to evaluate the effectiveness of the SHIN coating in preventing the migration of ions and/or electrons to or from the gold core, CV measurements were performed in an acidic media (0.1 M H_2_SO_4_). This would also test the robustness of the SHIN coating under electrode polarisation and resultant high electric fields. When electrochemically cycled in acidic media, the gold surface undergoes oxide formation and stripping during the anodic and cathodic sweeps, respectively. AuNPs are expected to exhibit this same behaviour, with oxidation and reduction peaks appearing on the cyclic voltammogram at around 1.2 V and 0.8 V *vs.* SCE respectively.^60^ If the SHIN coating is complete and pinhole free, the AuNP SHINs should not display these features due to the inaccessibility of the AuNP beneath.^28,61,62^

**Fig. 2:**
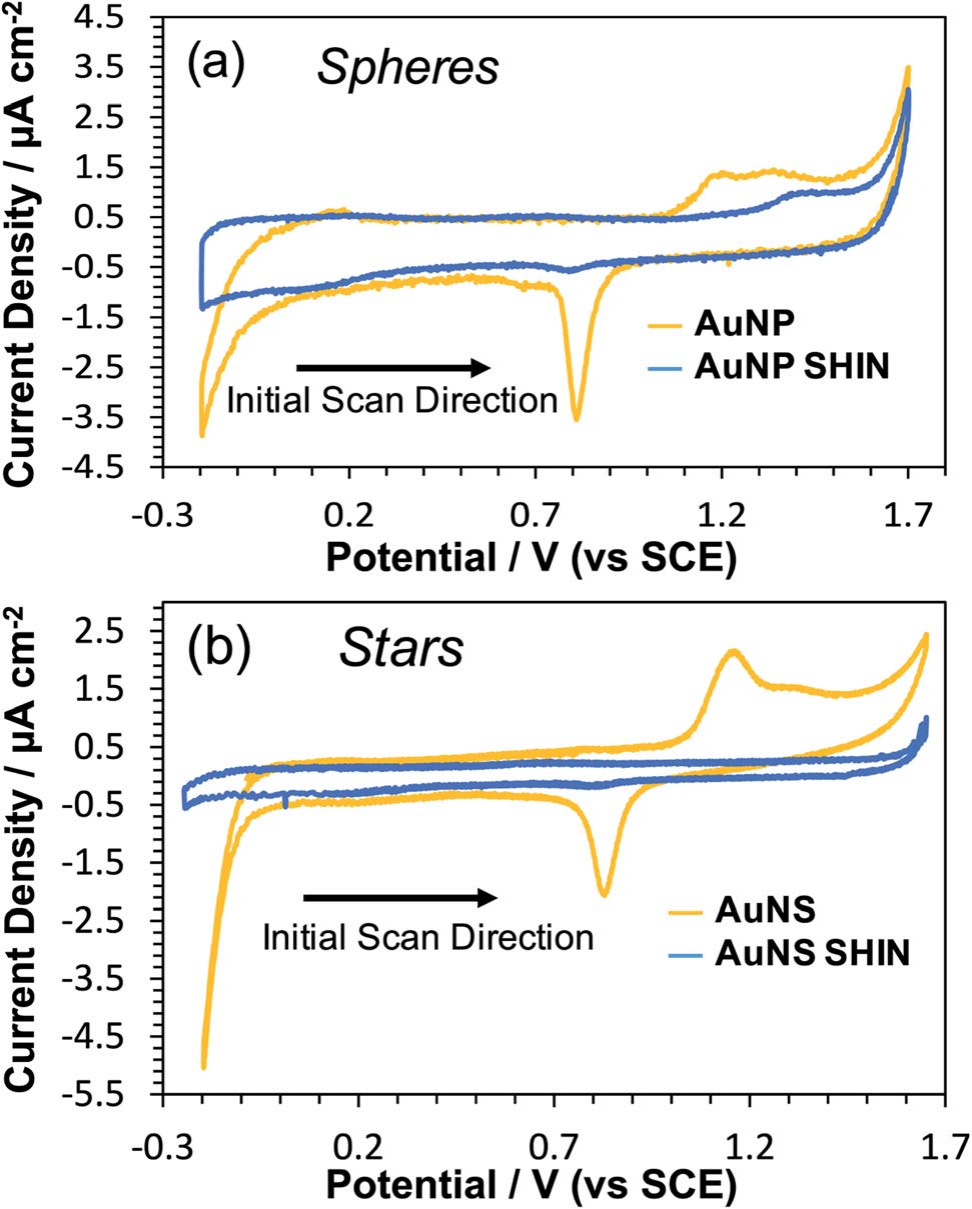
Cyclic voltammograms in 0.1 M H_2_SO_4_ comparing bare and SHIN coated nanoparticles deposited on separate ITO coated glass working electrodes: (a) AuNPs, (b) AuNSs. Scan rate was 50 mV s^−1^.

Fig. 2 shows comparative voltammograms for both bare AuNP and AuNP SHINs (Fig. 2(a)), in addition to AuNS and AuNS SHINs (Fig. 2(b)). For these experiments, a sub-monolayer of particles was electrostatically adsorbed onto the ITO working electrode surface. It is worth noting that the bare ITO exhibits a featureless background current in the absence of nanoparticles. An indication of the relatively high electrode fractional surface coverage of particles is shown in the dark-field images in Fig. S11.† While the Au oxidation and reduction peaks are not entirely supressed, particularly for the AuNP SHIN, the electrochemical potential window for these measurements is wide and it is clear that an effective barrier coating leading to strong passivation has been demonstrated (Fig. 2). This has also been demonstrated in previous SHINERS studies using SiO_2_ coatings, *e.g.* by Galloway *et al.*^61^ The voltammogram for AuNS SHINs shows superior passivation behaviour compared to that of the AuNP SHINs, in addition to a lower capacitive current. Differences in the particle morphology, fractional coverage of immobilised particles, as well as variations in the ITO working electrode resistivity may contribute to the observed differences. In comparison, in the only previous polymer-shell SHINERS study by Ye *et al.*,^28^ a large redox peak appearing at ∼1.2 V *vs.* SCE was reported which indicated that full passivation of the gold nanoparticle core had not been achieved.

### 3.4 SERS characterization

A series of both bulk colloid and confocal Raman measurements were performed to both characterise and demonstrate the utility of the synthesised SHIN particles. MB was primarily used as the model analyte as it has strong reversible redox activity and could furthermore enable the demonstration of *in situ* spectro-electrochemical confocal SERS mapping approaching single-SHIN particle spatial resolution. Additional bulk measurements were also performed using non-fluorescent analytes pyridine and 4,4′-BiPy to examine the passivation ability of the SHIN coating and the SHINERS performance.

#### Bulk SERS measurements

Initial measurements were aimed at confirming the passivating ability of the SHIN layers and, more specifically, to test for the presence of pinholes. By exposing the SHIN particles to pyridine, pinholes can be detected as molecular adsorption onto the underlying gold core surface would be observed *via* Raman peaks unique to adsorbed pyridine at 1010 cm^−1^ and 1035 cm^−1^.^63^ Fig. S12† shows comparative baseline corrected spectra of AuNP SHINs and bare AuNPs exposed to 5 mM pyridine, illustrating the effectiveness of the passivating layer on the pyridine-gold adsorption process. For bare AuNPs, the Raman peaks at 1010 cm^−1^ and 1035 cm^−1^ are intense and sharp while for the SHINs these peaks are almost entirely absent, suggesting that the pathway for pyridine molecules to adsorb to the gold core has been blocked. The peak present in the SHIN spectrum at 1100 cm^−1^ (in Fig. S12†) is associated with the outer PSS SHIN layer, with the aryl-S stretch^64^ being responsible for this peak. Notably, this peak is only observed at high laser powers (36 mW) and long integration times (60 s). We focused here on comparing SERS measurements of bare AuNPs and the final AuNP SHINs as the AuNP@MUA particles are stable only at high pH and the AuNP@MUA@PDDA particles have a positive surface charge. In both cases differences in the analyte adsorption behaviour would impede a quantitative comparison.

Further bulk SERS measurements were carried out using the non-fluorescent Raman analyte 4,4′ BiPy to determine whether the SERS enhancement afforded to bare AuNPs was still present in the AuNP SHINs. The 4,4′ BiPy probe molecule was chosen due to it having a relatively high Raman cross section compared to other small molecules,^65,66^ and so allow the greatest chance of detection. Fig. S13† shows comparative spectra of bare AuNPs and AuNP SHINs exposed to 1 mM 4,4′ BiPy. Although the SERS intensity for the characteristic peak of 4,4′ BiPy at ∼1010 cm^−1^ (ring breathing mode)^67,68^ on the AuNP SHINs was reduced by 63% compared to bare AuNPs, it is still clearly detectable. This SERS experiment was also repeated with the AuNS SHINs. Again here, the 4,4′ BiPy can be clearly detected by the AuNS SHINs from the characteristic peak at ∼1010 cm^−1^, as illustrated in Fig. S14 and S15.† The AuNS SHINs SERS intensity of that peak was significantly reduced (some 83% lower) compared to the bare AuNS. The greater reduction in SERS intensity for AuNS SHINs compared to AuNP SHINs for the 1010 cm^−1^ feature is likely to be due to the SHIN layers affecting both the number and accessibility of SERS hotspots generated. It is also likely that differences in the molecular adsorption behaviour (orientation and polarisation)^69,70^ and compactness are factors which could contribute to this, along with the separation distance between 4,4′ BiPy and the gold surface.

#### *In situ* SERS measurements on an electrode surface

To evaluate the SHIN nanoparticles for non-interfering enhanced Raman monitoring on electrode surfaces, spectro-electrochemistry measurements were performed in a custom *in situ* cell (see Fig. S2†) that also enabled high spatial resolution confocal Raman mapping. Measurements were performed using MB as the analyte, which was adsorbed on both bare and SHIN coated particles immobilised on an ITO working electrode.

A typical CV of the redox active MB analyte is shown in Fig. S16† which features oxidation and reduction peaks at −0.149 V and −0.172 V *vs.* SCE, respectively. MB is emissive at anodic potentials (oxidised state) and non-emissive at cathodic potentials (reduced state).^71^ MB also absorbs strongly at around ∼668 nm, thus making the molecule resonant with the chosen excitation laser, of 633 nm wavelength.^72^

**Fig. 3:**
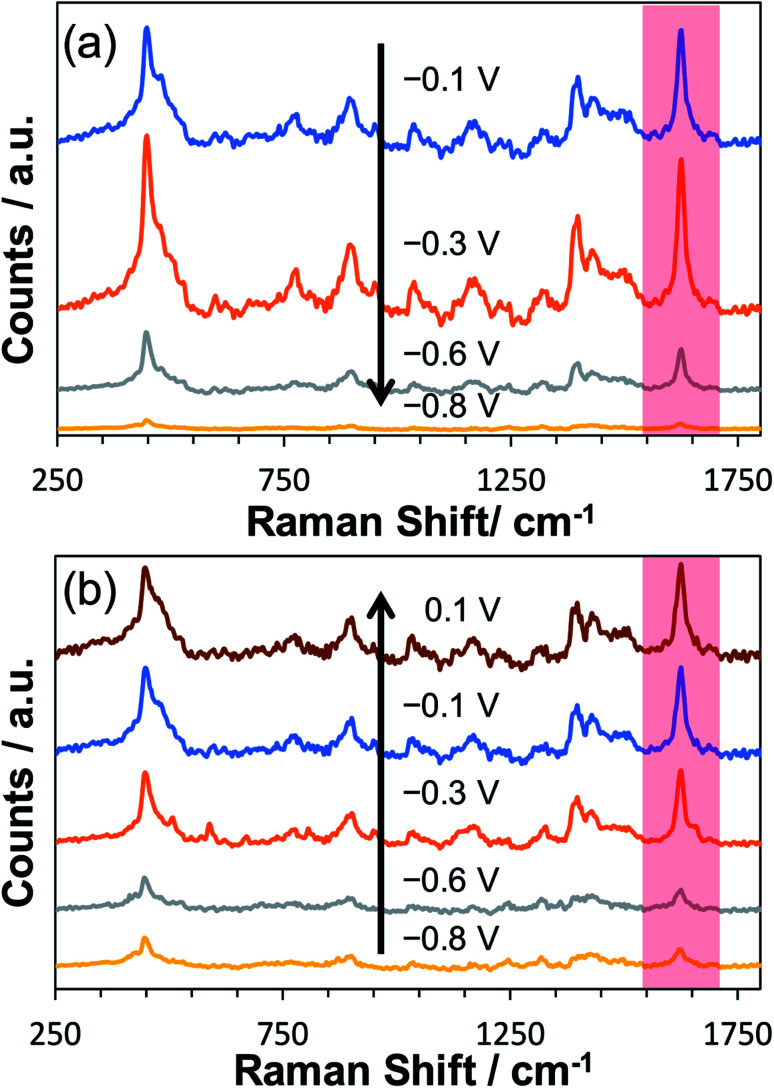
*In situ* SERS monitoring of a sub-monolayer of AuNP SHINs immobilised on an ITO working electrode which has been previously exposed to 0.1 μM MB. After rinsing the ITO surface and replacing with 0.1 M phosphate buffer (pH 5.6), the applied potential was (a) stepped from −0.1 V *vs.* Ag/AgCl to −0.8 V *vs.* Ag/AgCl and (b) stepped from −0.8 V *vs.* Ag/AgCl to 0.1 V *vs.* Ag/AgCl. After each step in potential, a dwell time of 2 minutes was implemented before SERS spectra were acquired. Laser wavelength was 633 nm and incident power was 6 μW with an integration time of 1 s. The peak of interest (1625 cm^−1^) is highlighted in red.

To explore the potential-dependent SERS behaviour of surface-adsorbed MB, an ITO working electrode decorated with AuNP SHIN particles was immersed in a 0.1 μM MB solution for 30 minutes. This procedure allowed physisorption of MB onto both the whole ITO surface and the AuNP SHINs. The ITO-AuNP SHIN surface was then rinsed and *in situ* spectro-electrochemistry was performed in a 0.1 M phosphate buffer (pH 5.6) electrolyte. Fig. 3 shows the relative changes in SERS spectra recorded as a function of the applied potential. As the potential was decreased from its open circuit value of −0.1 V *vs.* Ag/AgCl to −0.8 V *vs.* Ag/AgCl (Fig. 3(a)), a significant decrease in the SERS signal also occurred, corresponding to the reduction of the adsorbed MB to leuco-methylene blue (l-MB). On examining one characteristic peak of MB at ∼1625 cm^−1^ (attributed to the *ν*(C–C) ring stretch vibration)^73^ and looking at the data in Fig. 3(a) and (b), the peak intensity is substantially reduced at −0.8 V *vs.* Ag/AgCl compared to that at −0.1 V *vs.* Ag/AgCl but recovers close to its initial value as the potential is increased back to 0.1 V. It is worth noting that during this investigation of the potential dependence of the SERS signal, the laser was focused on the same spot.

A considerable challenge however was the reproducible measurement of SERS signals at each potential as laser thermal effects caused considerable photobleaching and spectral variation. Low laser powers were thus required to ensure minimal sample interference, and the spectra reported were averaged over 120 acquisitions. At the laser power (6 μW) employed here, no background Raman signal was observed from MB adsorbed on ITO only.

**Fig. 4:**
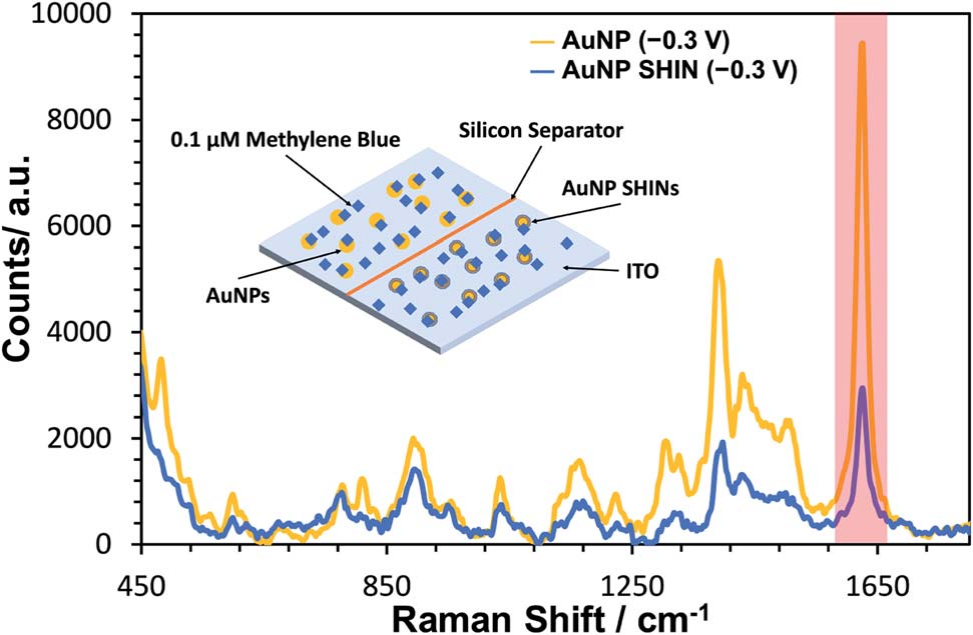
Comparison of *in situ* SERS spectra of bare AuNPs (gold trace) and AuNP SHINs (blue trace) immobilised side-by-side on an ITO working electrode which has been previously exposed to 0.1 μM methylene blue. The applied cell potential for both spectra was −0.3 V *vs.* Ag/AgCl. Laser wavelength was 633 nm and the laser power was 6 μW. Integration time was 1 s. An average was taken of 120 spectra collected in each area of the ITO surface to compensate for spectral variation. This was repeated in triplicate at random locations. The peak of interest (1625 cm^−1^) is highlighted in red.

A similar equivalent set of *in situ* measurements to that described in Fig. 3 was also performed for bare AuNPs, for which the same trend was observed (see Fig. S17†). In Fig. 4, SERS measurements were performed comparing both bare AuNPs and AuNP SHINs that were immobilized on separate areas of the same ITO electrode surface. The SERS spectra shown in Fig. 4 were acquired at the same applied potential of −0.3 V *vs.* Ag/AgCl, which corresponded to the maximum SERS signal response for the AuNP SHINs. As expected, there is a significant decrease in SERS intensity when comparing AuNPs to SHINs due to the SHIN coating causing a small increase in the distance between the plasmonic enhancer (gold core) and the analyte on the surface of the ITO electrode.^74^ A comparison of the peak intensities at MB's characteristic peak at ∼1625 cm^−1^ shows that the SERS intensity from the AuNP SHINs immobilised on the ITO surface is reduced by 68% compared to bare AuNPs. Despite this, given the low laser power (6 μW) and the short integration time (1 s) used, a high signal-to-noise still observed for the AuNP SHIN measurements indicates a strong enhancement of the SERS signal.

#### Confocal SERS surface mapping

While there are examples of *in situ* SERS spectro-electrochemical analysis of single particles/molecules^75–77^ and separate examples of single particle SERS mapping,^78–80^ there are no examples to date of a confocal single particle SHINERS mapping approach being combined with *in situ* spectro-electrochemistry that we are aware of.

**Fig. 5:**
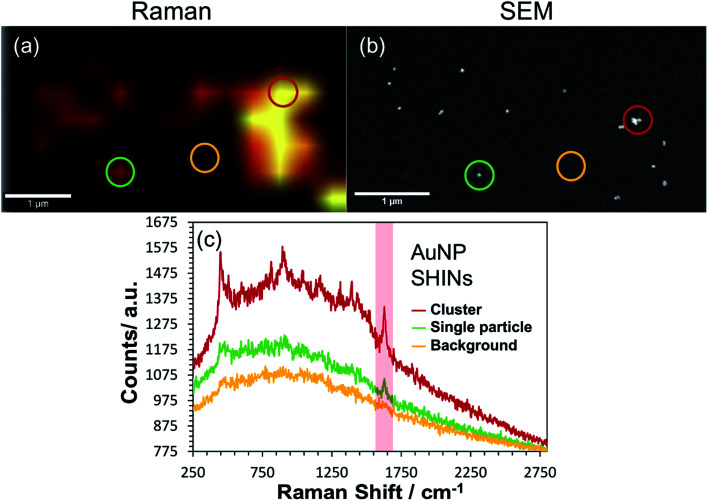
Correlated (a) confocal Raman and (b) SEM mapping of single AuNP SHINs immobilised on an ITO surface which has been previously exposed to 0.1 μM methylene blue. The scale bars in both images are 1 μm. The *ex situ* acquired Raman map was generated by plotting the difference in min–max intensities in the vicinity of the 1625 cm^−1^ peak as highlighted in the representative spectra (non-background corrected) shown in (c). Coloured circles on (a) and (b) correspond to regions where the representative spectra were obtained. Laser wavelength was 633 nm, laser power was 0.2 mW and each spectral point was acquired with an integration time of 1 s. Data was acquired at 0.4 μm spatial steps.

Correlated confocal Raman and SEM mapping was performed to confirm single particle SERS mapping can be achieved using the new SHIN design. To promote analysis at the single particle level, the ITO electrode surface was prepared with a relatively low surface particle coverage. Previous work has shown our approach to be valid for single particle confocal Raman mapping.^81^Fig. 5 presents results for AuNP SHIN particles immobilized on an ITO working electrode surface with adsorbed MB analyte molecules. There is excellent correlation between regions of high Raman scatter in the Raman map, Fig. 5(a), and individual particles in the SEM image, Fig. 5(b). The largest signals in the Raman map can be attributed to multiple SHINs within the laser focus while weaker signals can be assigned to spatially separated individual SHINs observed in the correlated SEM map. The representative spectra displayed in Fig. 5(c) of three distinct regions (indicated by circles) allows easy distinction between multiple and single particles, and background only areas. Both images in Fig. 5 were extracted from a larger mapped area which is shown in Fig. S18.†

**Fig. 6:**
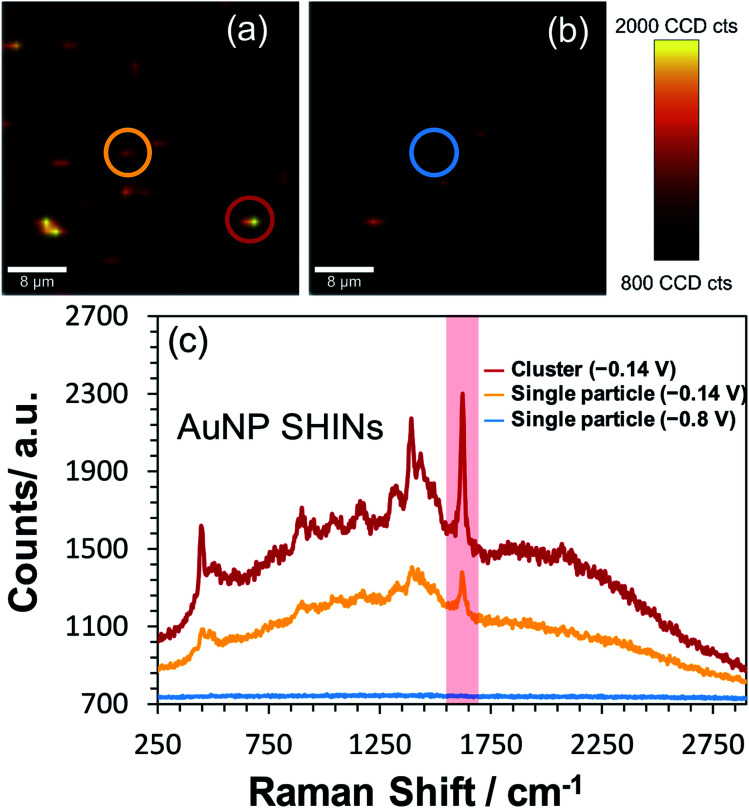
*In situ* confocal Raman mapping of AuNP SHINs immobilised on an ITO working electrode which has been previously exposed to 0.1 μM methylene blue. In (a) the potential is −0.14 V *vs.* Ag/AgCl (open circuit potential) and in (b) −0.8 V *vs.* Ag/AgCl is applied, with the same area mapped in each image. The scale bars in each map is 8 μm. Each map was generated by plotting the difference in min–max intensities in the vicinity of the 1625 cm^−1^ peak as highlighted in the representative spectra (non-background corrected) shown in (c). Circles on maps (a) and (b) correspond to regions where the spectra were obtained and shown in (c). Laser wavelength was 633 nm, laser power was 45 μW and each spectral point was acquired with an integration time of 0.5 s. Data was acquired at 1.25 μm spatial steps.

To test the effectiveness of the new SHIN design for elucidating localised structural features with single particle resolution, *in situ* confocal SERS mapping was performed. An ITO electrode surface was again prepared to promote low particle coverage and limit drying induced aggregation. Here, the same electrode area was mapped sequentially at the open circuit potential of −0.14 V *vs.* Ag/AgCl and at the applied potential of −0.8 V *vs.* Ag/AgCl. As both SERS maps in Fig. 6(a) and (b) are plotted using the same intensity scale associated with the 1625 cm^−1^ peak signal, the dramatic drop in SERS intensity at −0.8 V *vs.* Ag/AgCl indicates the reduction of MB to the non-emissive leuco-methylene blue, as observed previously in Fig. 3. The representative spectra presented in Fig. 6(c) compares data acquired from an isolated AuNP SHIN at the two potentials as well as showing the spectrum acquired from a different region yielding a brighter SERS signal. Similar to the *ex situ* study performed (Fig. 5), given the low excitation power and relatively short integration time employed for these measurements, the signal-to-noise achieved for MB is excellent. Interestingly, the SERS map in Fig. 6(b) still does indicate some locations where the MB molecules have not been completely reduced to leuco-methylene blue. A likely reason for this is that MB molecules adsorbed on the AuNP SHINs may be electrically isolated from the ITO surface and thus remain in the oxidised form even at −0.8 V *vs.* Ag/AgCl. It may well be that in these regions, the packing and passivating nature of the AuNP SHIN prevented electron tunnelling to the ITO substrate and so remained in the MB form.

**Fig. 7:**
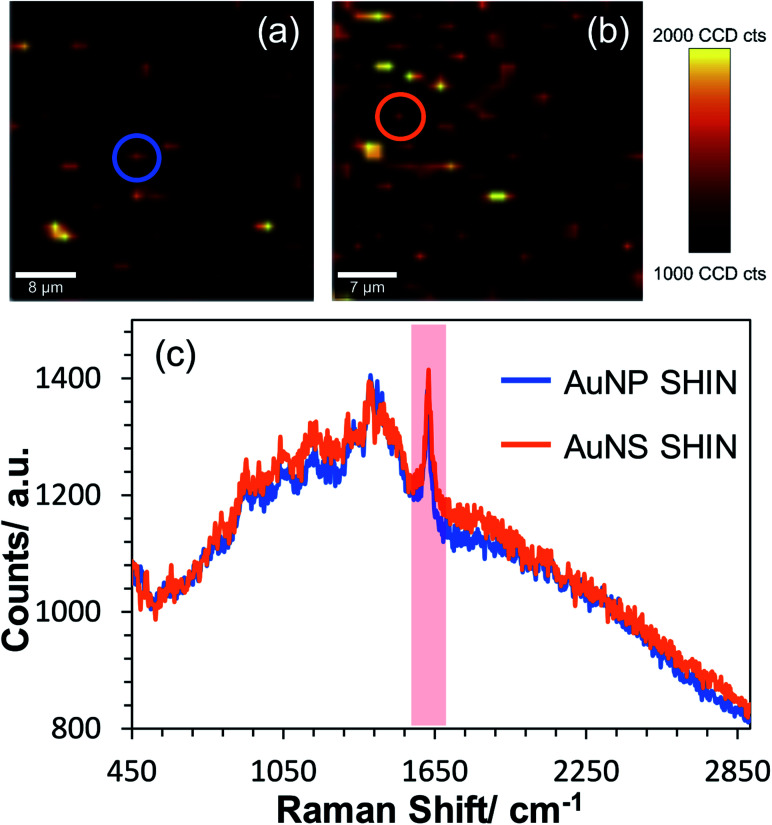
Confocal Raman mapping of (a) AuNP SHINs and (b) AuNS SHINs immobilised on an ITO working electrode which has been previously exposed to 0.1 μM methylene blue at the open circuit cell potential of −0.14 V *vs.* Ag/AgCl. The scale bars in (a) = 8 μm, and (b) = 7 μm. Each map was generated by plotting the difference in min–max intensities in the vicinity of the 1625 cm^−1^ peak as highlighted in the representative spectra (non-background corrected) shown in (c), with the locations in which the spectra were extracted indicated by the circles in each map. Laser wavelength was 633 nm, laser power was 45 μW and each spectral point acquired with an integration time of 0.5 s. Data acquired at 1.25 μm spatial steps.

Confocal *in situ* SERS mapping was also performed for the AuNS SHIN samples and compared directly to the AuNP SHINs on the same ITO surface. Fig. 7 shows SERS maps of AuNS SHIN and AuNP SHIN in the presence of adsorbed MB on an ITO electrode surface. Both maps were recorded at the open circuit potential of −0.14 V *vs.* Ag/AgCl and were plotted on the same intensity scale associated with the 1625 cm^−1^ peak signal. The representative spectra in Fig. 7(c) indicate that both particles present a very similar spectral response with excellent signal-to-noise for both SHIN particles, demonstrating good repeatability across each particle shape. The representative spectra extracted from the circled regions in SERS maps Fig. 7(a) and (b) and plotted in Fig. 7(c) are weaker signals associated with isolated, individual particles (a similar signal intensity was observed in Fig. 5(c) also at −0.14 V *vs.* Ag/AgCl). This suggests that the SERS enhancement is not significantly different between the AuNP and AuNS SHINs at the single nanoparticle level.

## Conclusion

In this work, we fabricated a novel SHIN design consisting of passivating layers of MUA, PDDA, and PSS encapsulating AuNP and AuNS core shapes. Characterisation of the new SHINs indicated that the AuNP and AuNS cores could be synthesised with little polydispersity and that the prepared SHIN layers physically isolated the cores from exterior redox interactions. A highlight of the SHIN design is the flexibility of the surface chemistry, as well as the thickness and number of passivating layers through the LbL approach compared to conventional SHIN designs. We have also previously demonstrated the displacement of the CTAB surfactant monolayer surrounding gold nanorods with MUA followed by a polyelectrolyte layer,^82^ highlighting the potential for other shapes and surface chemistries beyond that described here. Furthermore, the SERS enhancement associated with the AuNP and AuNS SHINs was sufficient to enable one of the first reported studies of high resolution *in situ* confocal SERS mapping of SHINs on an electrode surface. Further work will involve directly comparing the SHIN coating *versus* more established silica coatings in terms of Raman performance and also different application scenarios. The visualisation of redox-related structural changes using the non-interfering SHIN design presented here has the potential for a wide range of applications in electroactive environments, such as the SERS mapping of battery and electrocatalytic surfaces approaching single nanoparticle spatial resolution. Beyond SERS, SHIN particles are also being increasingly applied to diverse areas such as biosensing and nanoelectronics, further opening up new opportunities which may potentially benefit from the improved design flexibility and control demonstrated in this work.

## Author contributions

DKB performed the experiments with contributions also from PAM and CWB. Data analysis, concept development and preparation of the manuscript all involved DKB, AJW, LEAB and AWW.

## Conflicts of interest

There are no conflicts to declare.

## Supplementary Material

NA-003-D1NA00473E-s001
