# Supplementary material for: A universal polymer shell-isolated nanoparticle (SHIN) design for single particle spectro-electrochemical SERS sensing using different core shapes
Source: Nanoscale Adv. 2021 Sep 21;3(22):6415–26. doi: 10.1039/d1na00473e (PMC9416900; doi:10.1039/d1na00473e)
Supplement: NA-003-D1NA00473E-s001 [file NA-003-D1NA00473E-s001.pdf]

Electronic Supplementary Material (ESI) for Nanoscale Advances.
This journal is © The Royal Society of Chemistry 2021

Electronic Supporting Information for:

A universal polymer shell-isolated particle (SHIN) design for single particle spectro-electrochemical SERS sensing using different core shapes

Delali K. Boccorh[a], Peter A. Macdonald[a], Colm W. Boyle[a], Andrew J. Wain[b], Leonard E.A. Berlouis[c], and Alastair W. Wark*[a]

[a] *Centre for Molecular Nanometrology, Technology and Innovation Centre, Dept. of Pure & Applied Chemistry, University of Strathclyde, 99 George St, Glasgow, UK, G1 1RD*

[b] *National Physical Laboratory, Hampton Road, Teddington, UK, TW11 0LW*

[c] *Dept. of Pure & Applied Chemistry, University of Strathclyde, 295 Cathedral St, Glasgow, UK, G1 1XL*

***Corresponding author:** Email: alastair.wark@strath.ac.uk, Tel: +44 (0)141 548 3084, ORCID:* orcid.org/0000-0001-8736-7566, *ResearcherID:* O-5400-2014

Contents

Additional supporting data figures:

Fig. S1	UV-Vis spectrum of gold citrate nanoparticle seed solution.
Fig. S2	Schematic of spectro-electrochemical cell used for SERS analysis.
Fig. S3	Cyclic voltammograms of PDDA and PSS solutions.
Fig. S4	Cyclic voltammograms of a polished bulk Au electrode coated with successive monolayers of the SHIN layers.
Fig. S5	Additional SEM images of AuNP and AuNP SHIN.
Fig. S6	Additional SEM images of AuNS and AuNS SHIN.
Table S1	Characterization summary of AuNP SHIN formation steps.
Fig. S7	Nanoparticle tracking analysis of each SHIN synthesis stage.
Table S2	Characterization summary of AuNS SHIN formation steps.
Fig. S8	UV-Vis spectra of multiple SHIN fabrications on the same AuNP core.
Fig. S9	UV-Vis spectra of repeat SHIN fabrication.
Fig. S10	UV-Vis spectra of AuNS bare and SHIN samples comparing stability.
Fig. S11	Optical darkfield images of ITO slide coated with nanoparticles.
Fig. S12	Bulk SERS Raman spectra of AuNPs and SHINs exposed to 5 mM pyridine.
Fig. S13	Comparison of bulk Raman spectra of AuNPs and AuNP SHINs exposed to 1 mM 4,4 BiPy.
Fig. S14	Comparison of bulk Raman spectra of AuNS and AuNS SHIN exposed to 1 mM 4,4 BiPy.
Fig. S15	Comparison of bulk Raman spectra of AuNP SHIN and EtOH
Fig. S16	Cyclic voltammogram of methylene blue.
Fig. S17	*In situ* SERS monitoring of a sub-monolayer of AuNPs immobilised on an ITO working electrode which has been exposed to 0.1 μM methylene blue.
Fig. S18	Correlated confocal Raman and SEM mapping of AuNP SHINs immobilised on an ITO working electrode which has been exposed to 0.1 μM methylene blue.

Additional Supporting Data

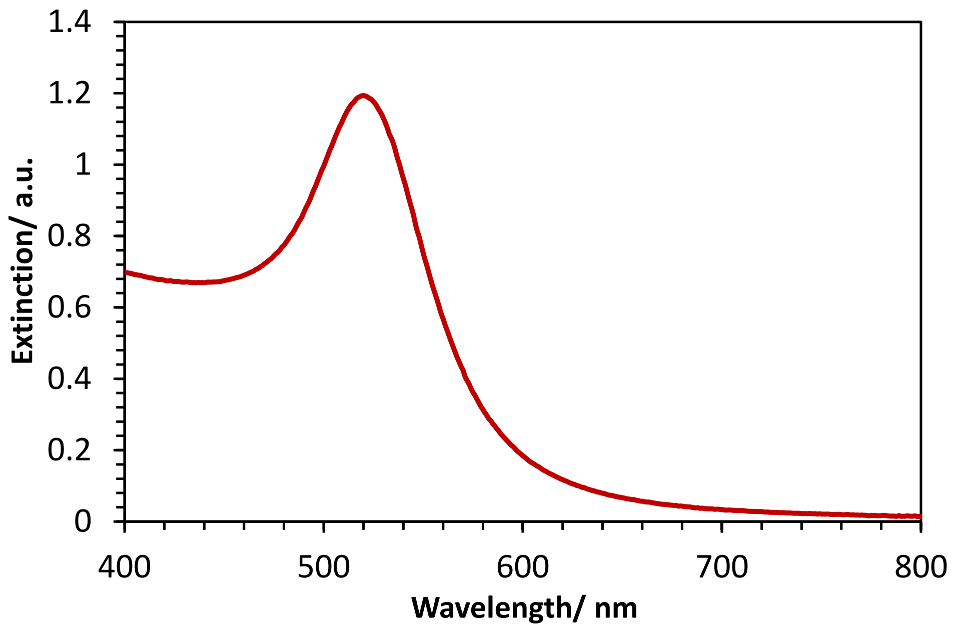

Fig. S1 *UV-Vis spectrum of gold citrate nanoparticle seed solution used for both larger AuNP and AuNS production.*

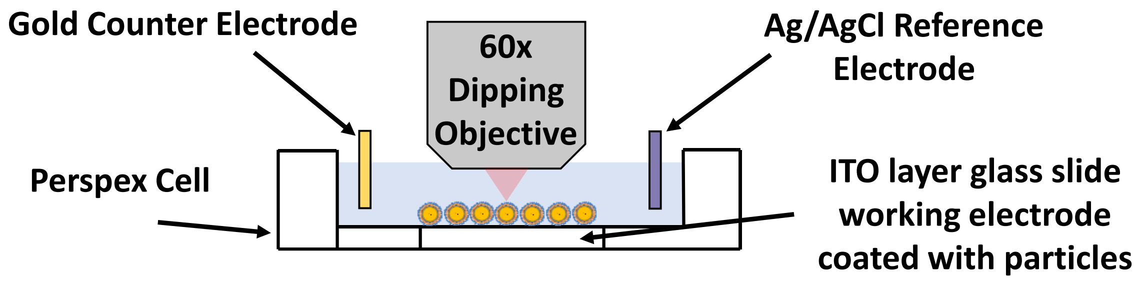

Fig. S2 *Schematic of spectro-electrochemical cell used for SERS analysis. The cell material was Perspex in which an ITO coated glass slide was fixed. The counter electrode was gold, and the reference electrode was Ag/AgCl. Raman scatter from working electrode surface was collected with a Nikon NIR Apo 60× (NA 1.0) water dipping objective.*

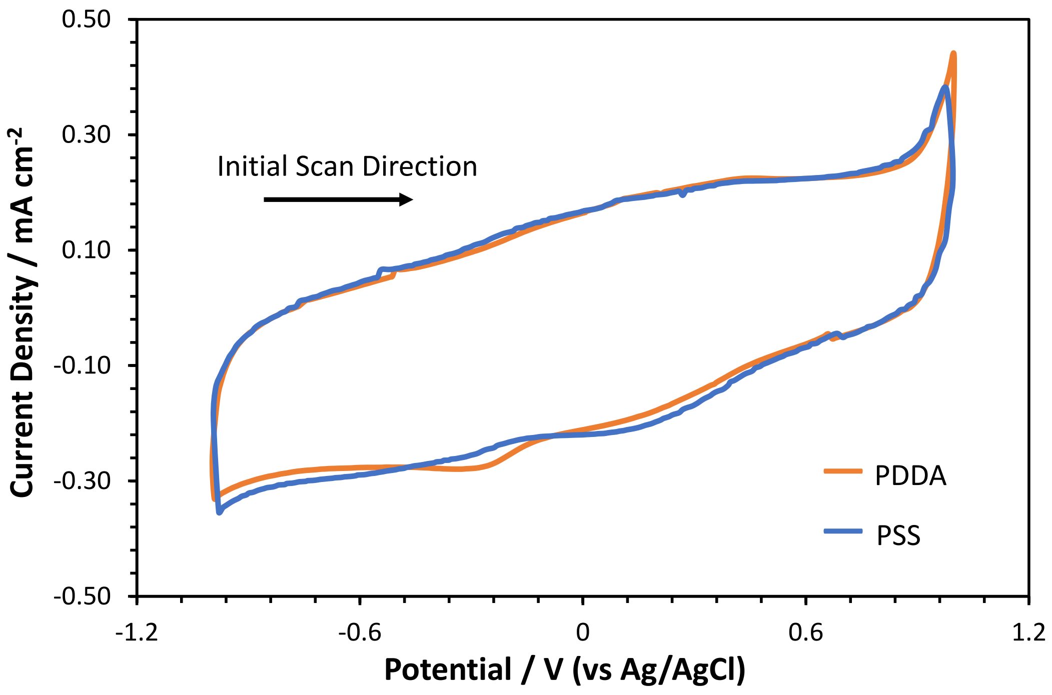

Fig. S3 *Cyclic voltammograms of separate ITO coated glass working electrodes, with PDDA, and PSS adsorbed onto their surfaces. Experiments were carried out in 0.5 M KCl electrolyte with a Pt counter electrode, and a Ag/AgCl reference electrode at a scan rate of 50 mV/s.*

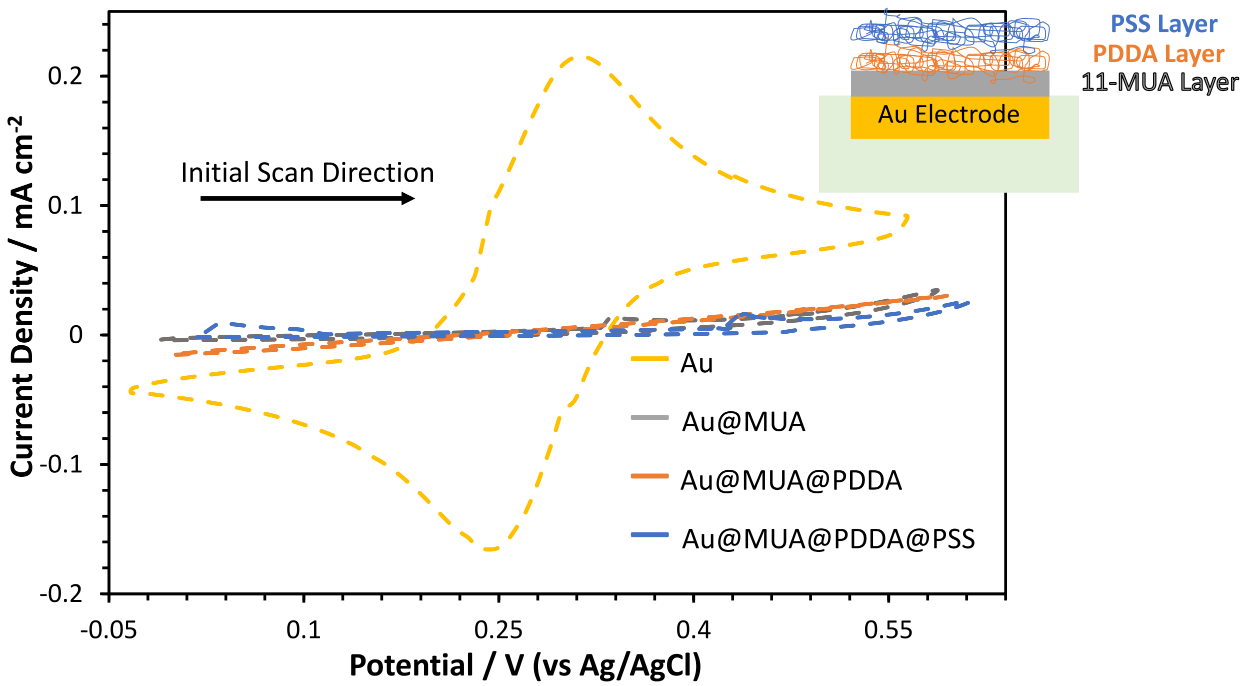

Fig. S4 *Cyclic voltammograms of a polished bulk Au electrode coated with successive monolayers of the SHIN layers: to form Au@MUA, Au@MUA@PDDA, and Au@MUA@PDDA@PSS electrodes. Experiments were carried out with a Au working electrode with a geometric surface area of 0.38 cm^2, Pt counter electrode, and a Ag/AgCl reference electrode at a scan rate of 50 mV/s in 1 mM $K_4[Fe(CN)_6]$ in 0.1 M KCl.*

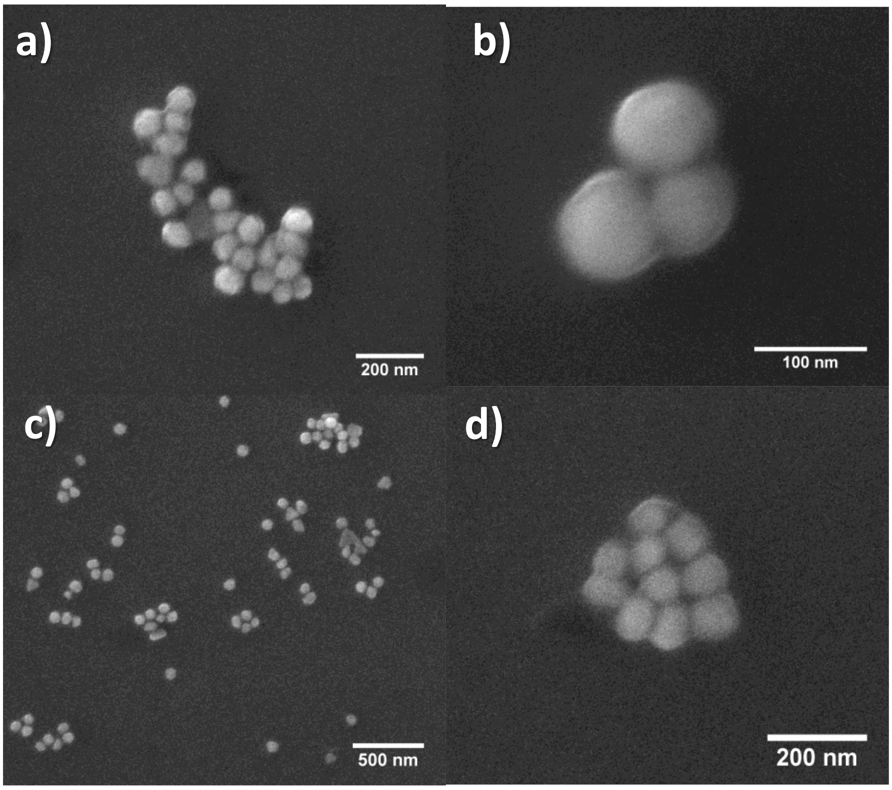

Fig. S5 *Additional SEM images of a) and b) bare quasi-spherical AuNPs, and c) and d) AuNP SHINs.*

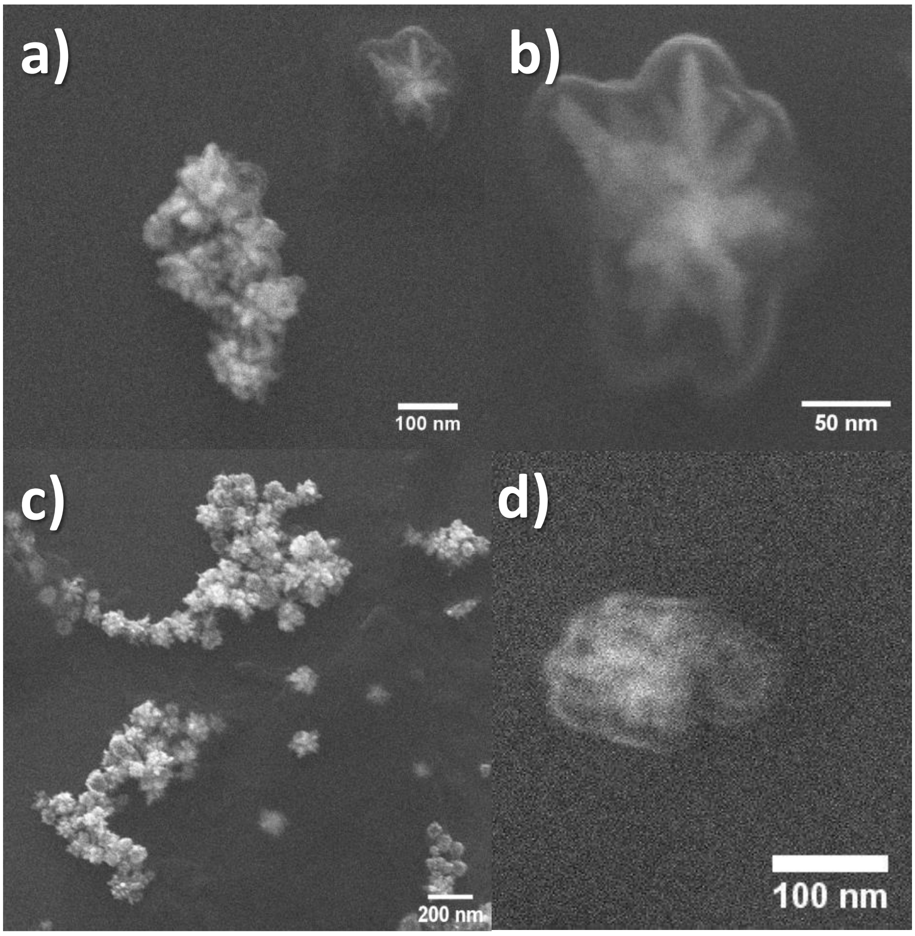

Fig. S6 *Additional SEM images of a) and b) bare AuNS, and c) and d) AuNS SHINs.*

Table S1 *Characterization summary of AuNP SHIN formation steps.*

**Sample**	**λ_{max} / nm**	**Zeta Potential / mV**	**NTA Size/ nm**	**SEM Size/ nm**
AuNP	545	– 44.1 (± 10.9)	89 (± 3)	84 (± 9)
AuNP@MUA	549	– 35.8 (± 16.9)	94 (± 3)	N/A
AuNP@MUA@PDDA	549	49.6 (± 13.8)	89 (± 2)	N/A
AuNP SHIN (AuNP@MUA@PDDA@PSS)	549	– 68.8 (± 19.1)	88 (± 1)	88 (±10)

Errors were calculated by determining the standard error (SE) of each sample population. For zeta potential and NTA, this was achieved by using the standard deviation (SD) generated by the acquisition and analysis software to then calculate the SE. For SEM, determining the SE was achieved by first manually calculating the SD from a large sample population before calculating the SE.

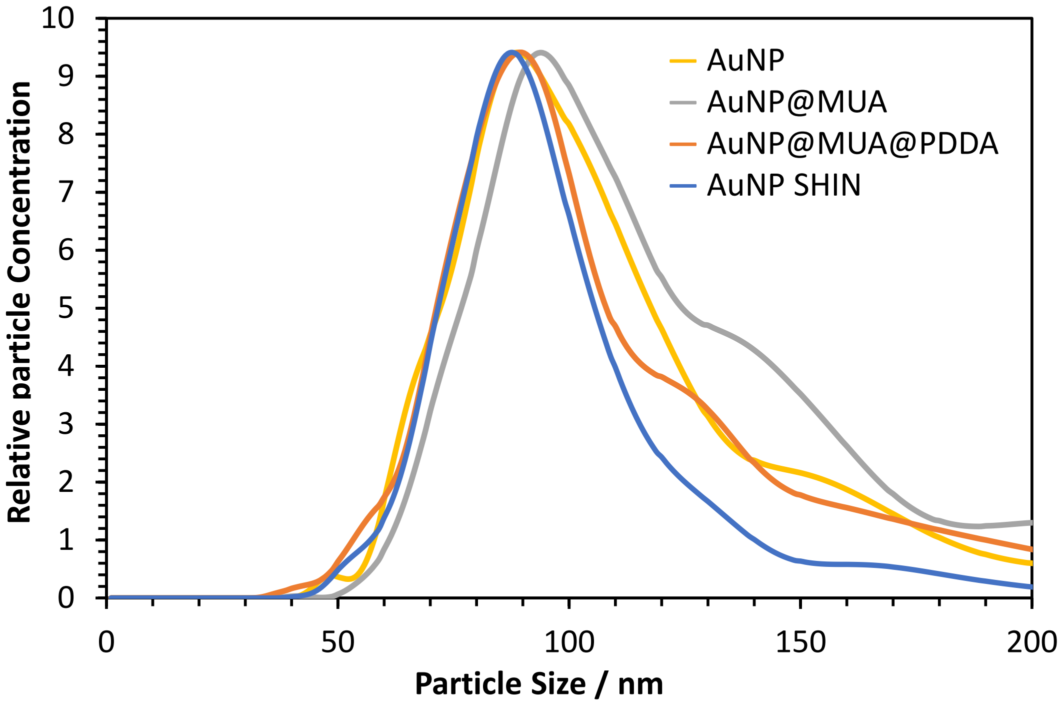

Fig. S7 *Nanoparticle Tracking Analysis of each SHIN synthesis stage on ~80 nm AuNPs core. Each trace has been normalised with respect to the relative concentration maximum for comparison. Each sample stock was diluted by a factor of 100 before analysis.*

Table S2 *Characterization summary of AuNS SHIN formation steps.*

Sample	λ_{max} / nm	Zeta Potential / mV	SEM Size/ nm
AuNS	790	−35.5 (±11.8)	121 (± 21)
AuNS@MUA	816	−65.1 (±15.9)	N/A
AuNS@MUA@PDDA	839	49.5 (±10.4)	N/A
AuNS SHIN (AuNS@MUA@PDDA@PSS)	814	−32.4 (±13.7)	124 (± 31)

Errors were calculated in the same way detailed in Table S2.

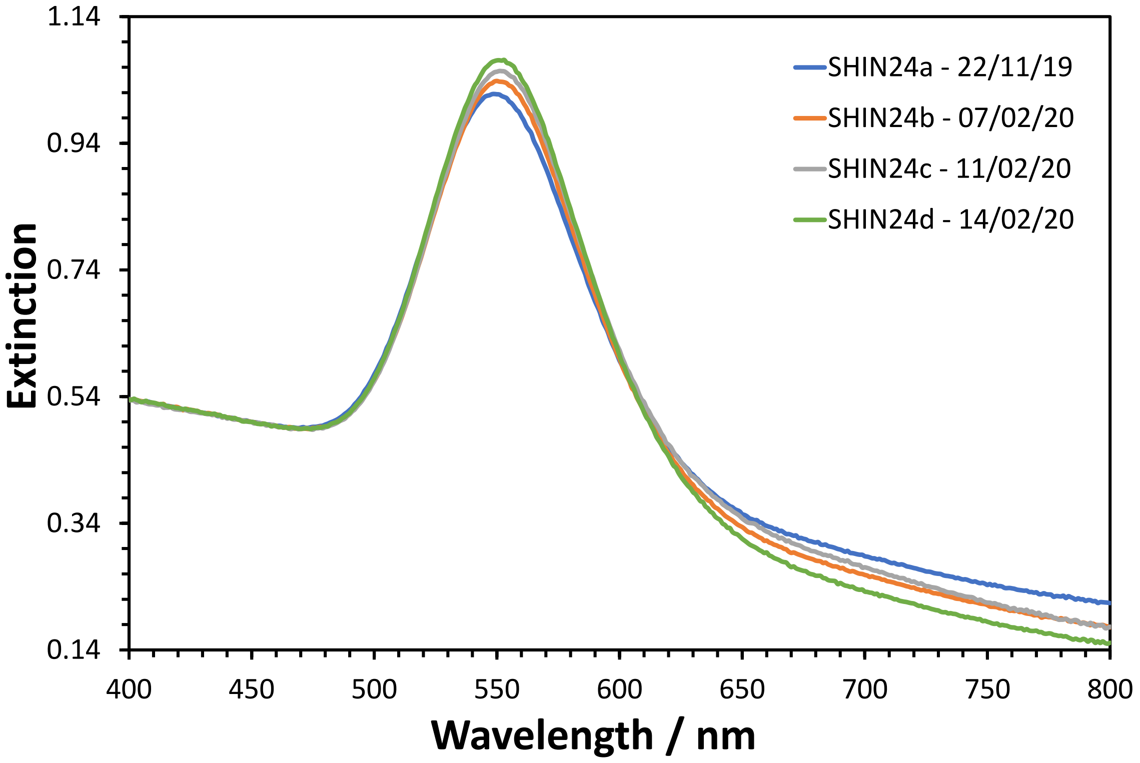

Fig. S8 *UV-Vis spectra of multiple repeat AuNP SHIN fabrications using the same AuNP core stock solution to highlight coating reproducibility across batches. The slight difference between the 24a batch compared to the very similar 24b, 24c and 24d batches is attributed to a change in the AuNP stock after several weeks of storage. Spectra are normalized at 450 nm for comparison.*

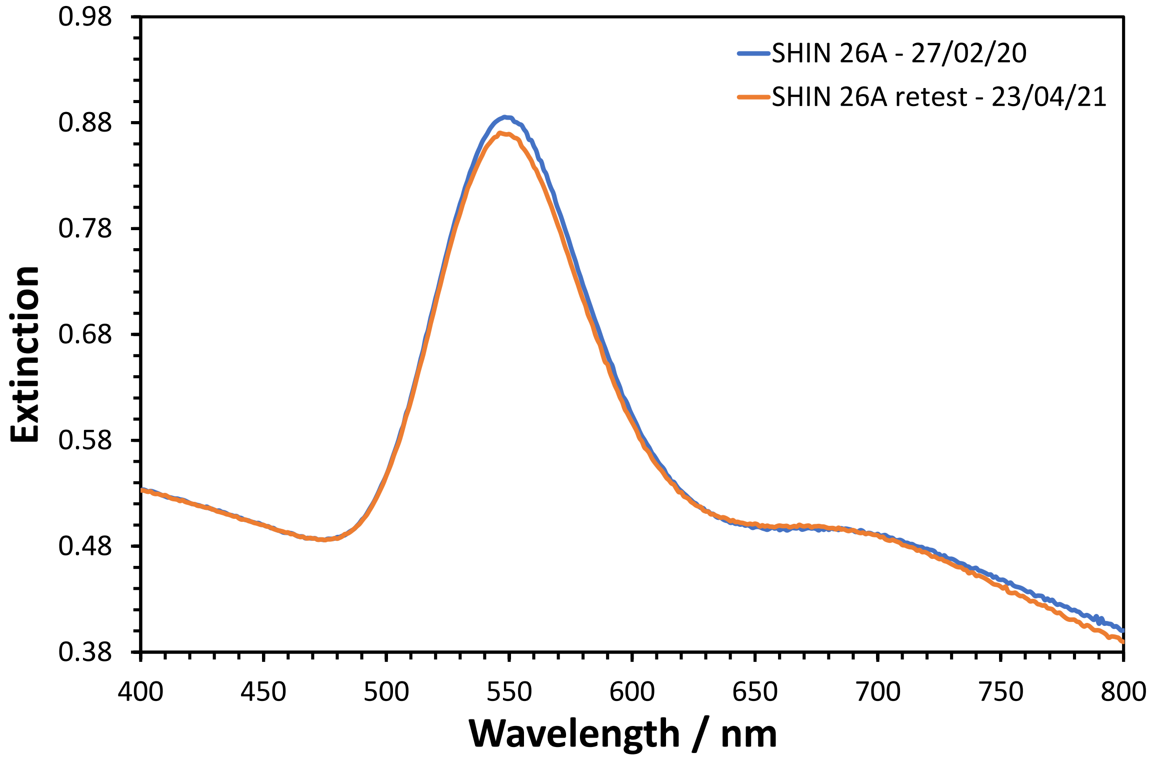

Fig. S9 *UV-Vis spectra of the same AuNP SHIN sample acquired 14 months apart taken to highlight particle stability over time. Spectra are normalized at 450 nm for comparison.*

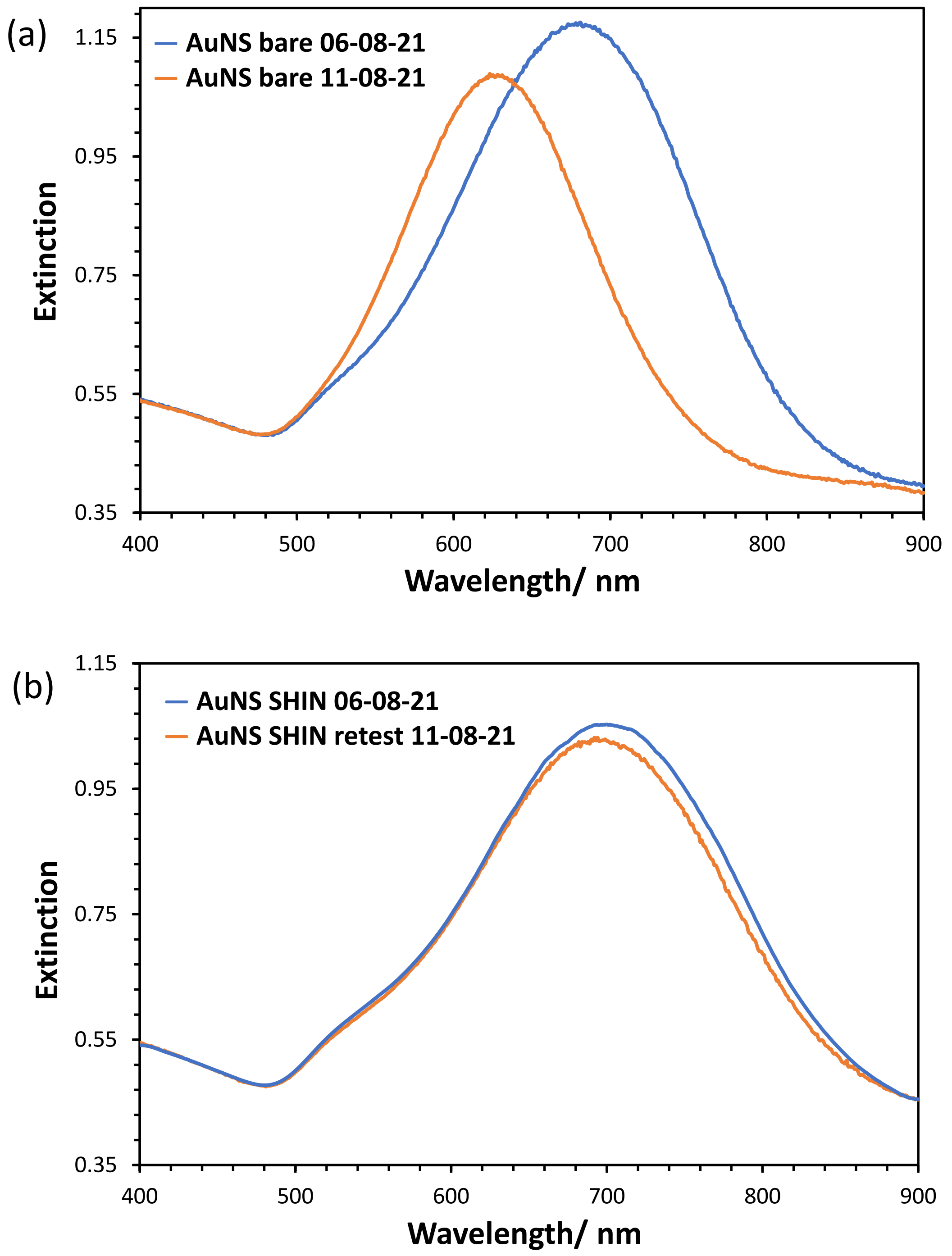

Fig. S10 *UV-Vis spectra of (a) a AuNS stock solution on their preparation and after only 5 days of storage. (b) The corresponding AuNS SHIN prepared from the same stock and stored for 5 days. It can be clearly seen that the AuNS stock significantly blue shifts while SHIN sample is much more stable. Further long-term studies for different NS morphologies are needed.*

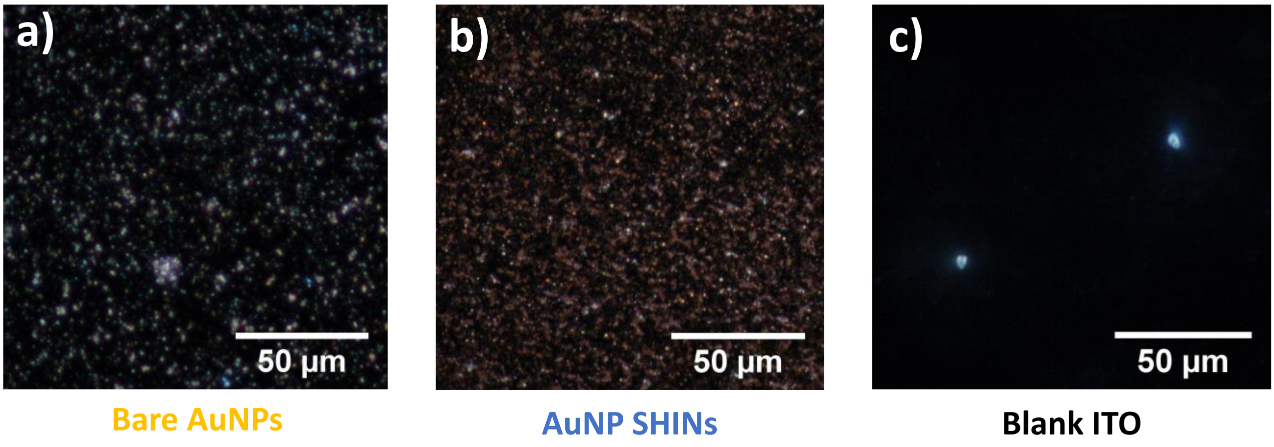

Fig. S11 *Optical darkfield images of: a) Uncoated AuNPs immobilised on an ITO slide, b) AuNP SHINs immobilised on an ITO slide, and c) a blank ITO coated slide for comparison with reference to the data in Figure 2 in the main article. Each image was acquired under identical light intensity and camera integration times to demonstrate differences in surface scattering morphology.*

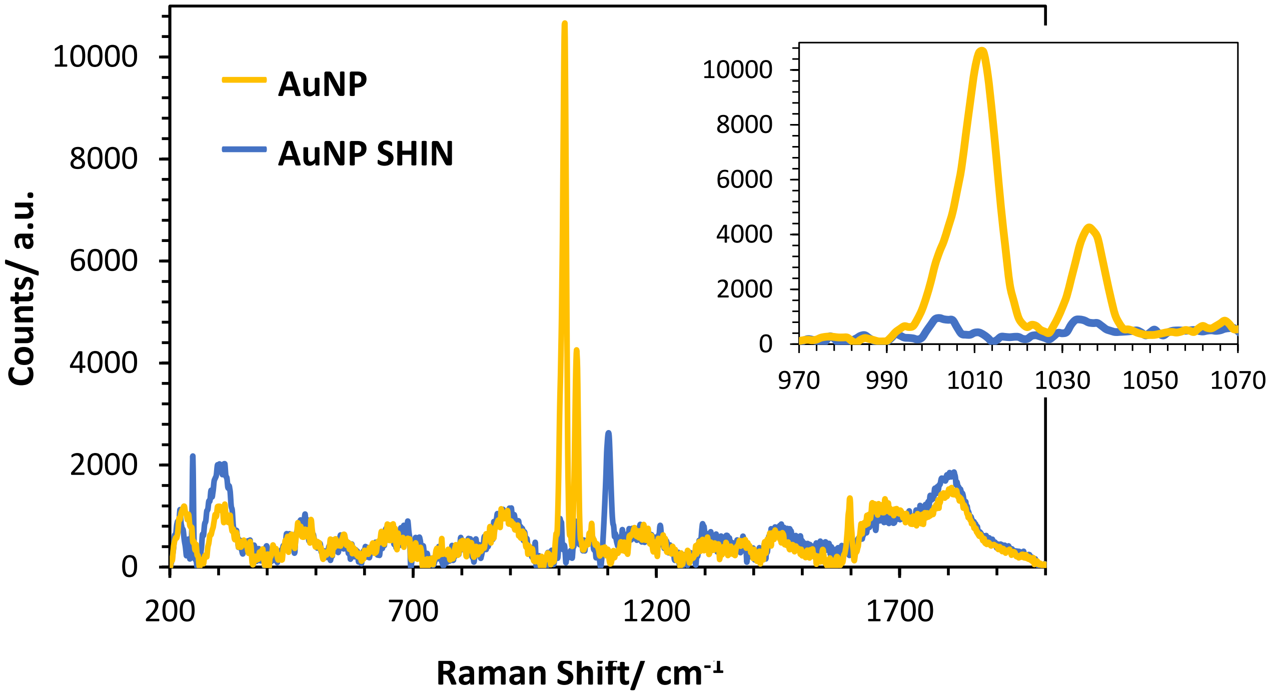

Fig. S12 *Bulk SERS Raman spectra of AuNPs and SHINs exposed to 5 mM pyridine with the figure inset focussing on SERS peaks at 1010 cm^{-1} and 1035 cm^{-1}. Laser wavelength was 638 nm, and the max laser power was 36 mW. Integration time was 60 s. Spectra were baseline corrected. Measurements were carried out on a Snowy Range Instruments (SnRI) Sierra series 638 nm Raman spectrometer.*

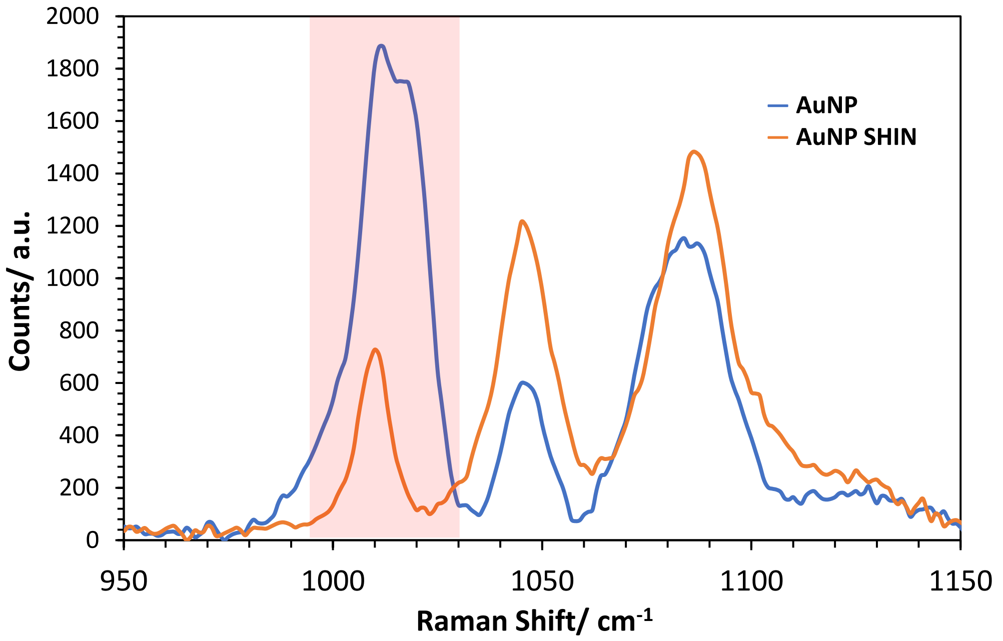

Fig. S13 *Comparison of bulk Raman spectra of AuNPs (blue trace) and AuNP SHINs (orange trace) exposed to 1 mM 4,4′ BiPy dissolved in 100 mM EtOH. Laser wavelength was 785 nm and the laser power was 78 mW. Integration time was 10 s. Measurements were carried out on a Snowy Range Instruments (SnRI) Sierra series 785 nm Raman spectrometer. Spectra have been baseline corrected. Note due to the presence of EtOH only the highlighted peak at 1110 cm^{-1} is reliable for comparison purposes (see Fig. S15).*

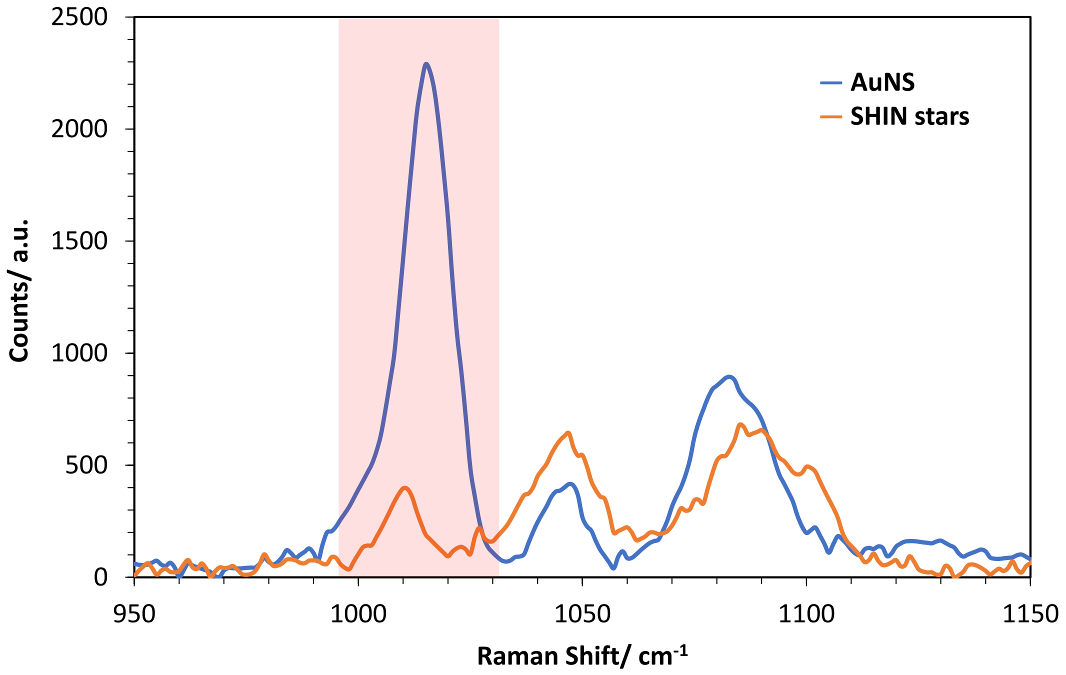

Fig. S14 *Comparison of bulk Raman spectra of AuNS (blue trace) and AuNS SHIN (orange trace) exposed to 1 mM 4,4′ BiPy dissolved in 100 mM EtOH. Laser wavelength was 785 nm and the laser power was 78 mW. Integration time was 10 s. Measurements were carried out on a Snowy Range Instruments (SnRI) Sierra series 785 nm Raman spectrometer. Spectra have been baseline corrected. Note due to the presence of EtOH only the highlighted peak at 1110 cm^{-1} is reliable for comparison purposes (see Fig. S15).*

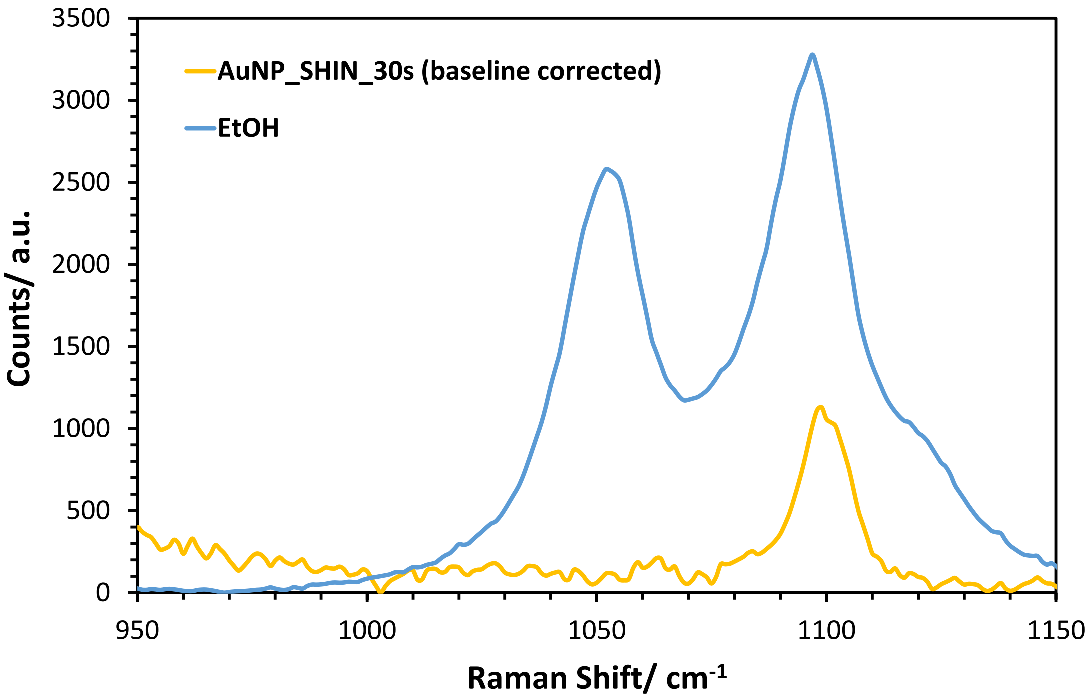

Fig. S15 *Comparison of bulk Raman spectra of AuNP SHIN (yellow trace) and EtOH (blue trace). Laser wavelength was 785 nm and the laser power was 78 mW. Integration time was 30 s. Measurements were carried out on a Snowy Range Instruments (SnRI) Sierra series 785 nm Raman spectrometer. Spectra have been baseline corrected. These results show that the presence of EtOH in which the BiPy stock solution is prepared befosre diluting in water masks the 1100 cm^{-1} PSS peak but not the 1010 cm^{-1} BiPy peak.*

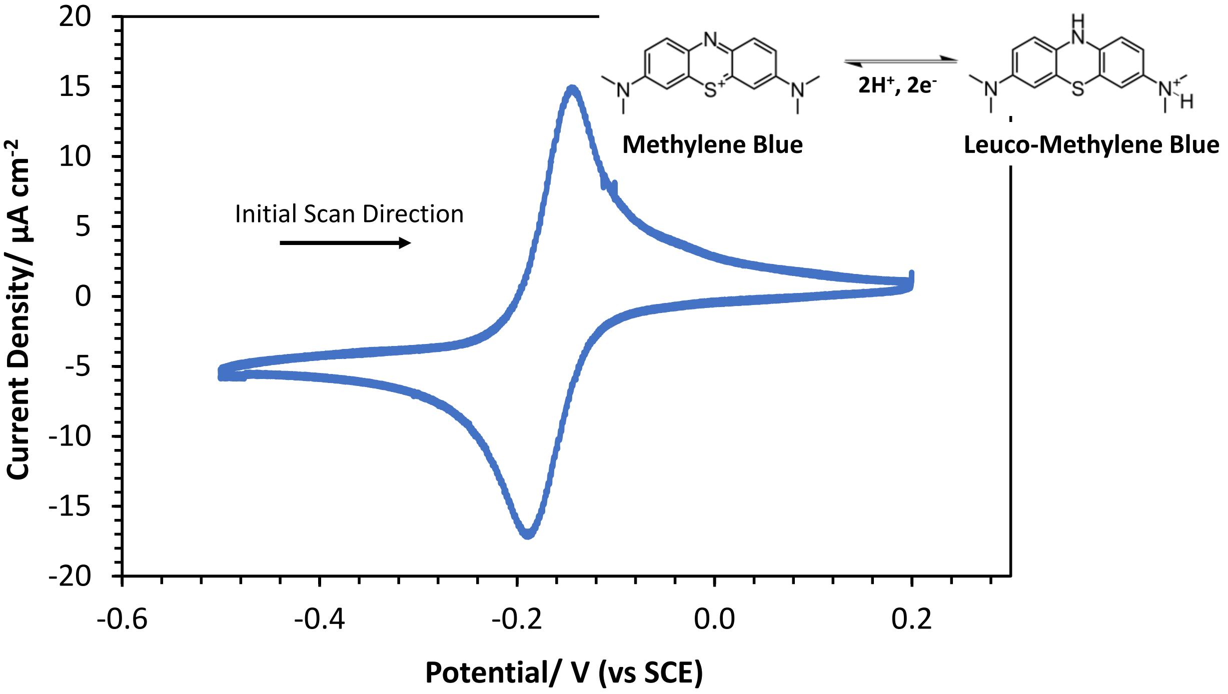

Fig. S16 *Cyclic voltammogram of MB collected by immersing an ITO glass slide in a 0.1 mM solution of MB in pH 5.6 phosphate buffer. Scan rate was 20 mV/s.*

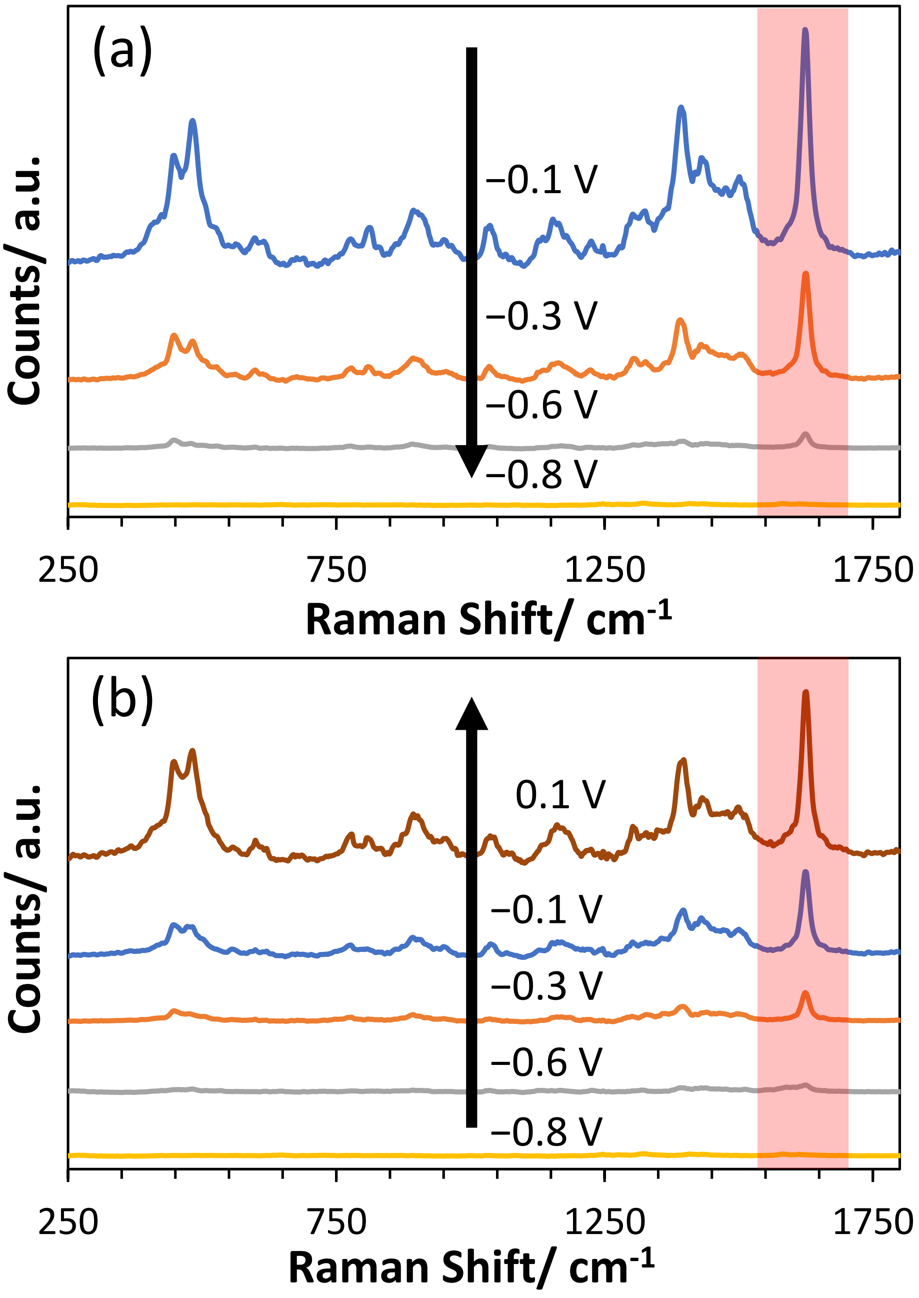

Fig. S17 *In-situ SERS monitoring of a sub-monolayer of AuNPs immobilised on an ITO working electrode which has been exposed to 0.1 µM MB. After rinsing the ITO surface and replacing with 0.1 M phosphate buffer (pH 5.6), the applied cell potential was stepped from a starting potential of −0.1 V vs Ag/AgCl and held at intervals initially in a) negative potential direction and then (b) subsequently reversed. Laser wavelength was 633 nm and incident power was 6 µW with an integration time of 1 s. Peak of interest (1625 cm^{-1}) is highlighted in red.*

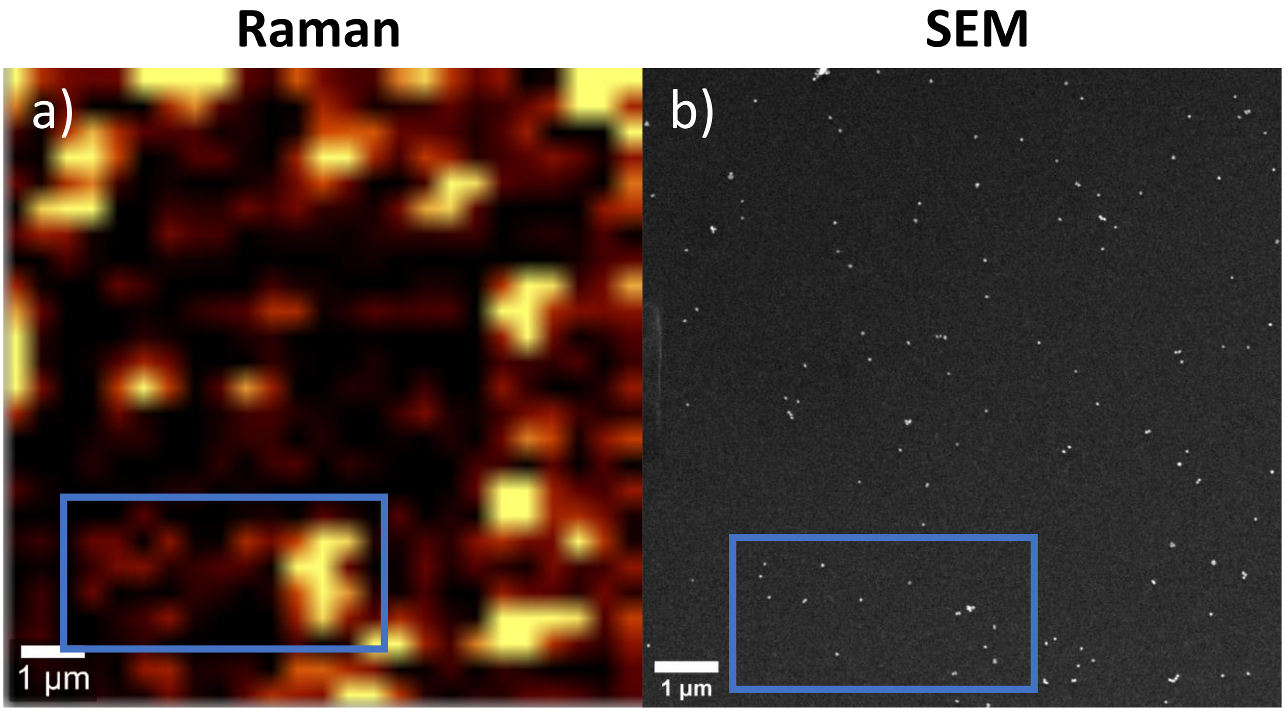

Fig. S18 *Expanded version of the correlated Raman and SEM map shown in Fig. 5 of the main article with the regions presented indicated by the coloured rectangles. Acquisition details are described in the Fig. 5 caption and the experimental section of the main article. Scale bars are equal to 1 micron in both images.*
